# Coordination of leaf function and carbohydrate reserves is associated with cultivar differences in mango (*Mangifera indica*) fruit productivity

**DOI:** 10.1093/aob/mcag188

**Published:** 2026-06-27

**Authors:** Gerhard C Rossouw, Bruno R Tamelini, Carole Wright, Geoffrey Dickinson, Ryan Orr

**Affiliations:** Queensland Department of Primary Industries, Mareeba Research Facility, Mareeba, Queensland 4880, Australia; Queensland Department of Primary Industries, Mareeba Research Facility, Mareeba, Queensland 4880, Australia; Queensland Department of Primary Industries, Mareeba Research Facility, Mareeba, Queensland 4880, Australia; Queensland Department of Primary Industries, Mareeba Research Facility, Mareeba, Queensland 4880, Australia; Queensland Department of Primary Industries, Mareeba Research Facility, Mareeba, Queensland 4880, Australia

**Keywords:** *Mangifera indica*, carbohydrate reserves, leaf physiology, carbon allocation, fruit productivity, planting density, source–sink relationships, orchard intensification

## Abstract

**Background and Aims:**

Optimizing fruit tree productivity largely depends on physiological traits that regulate carbon acquisition and non-structural carbohydrate (NSC) partitioning. In mango (*Mangifera indica*), the contribution of NSC reserves and leaf functionality to fruit development under intensified orchard systems remains poorly understood. This study provides the first integrated, seasonally resolved comparison of leaf function and NSC dynamics between mango cultivars differing in productivity, identifying physiological traits associated with yield and NSC use efficiency.

**Methods:**

‘Keitt’ (higher-yielding) and ‘Yess!’ (lower-yielding) trees, previously established under both lower (208 trees ha^−1^) and higher (1250 trees ha^−1^) planting densities, were monitored over two consecutive fruiting seasons. Leaf carbon assimilation-associated traits were measured, including stomatal conductance, stomatal morphology, chlorophyll content, stable carbon isotopes (δ^13^C) and NSC concentrations. Additionally, NSC reserves in roots and trunks were quantified across key phenological stages to evaluate patterns of reserve replenishment and mobilization. These physiological metrics were then related to fruit yield and yield efficiency.

**Key Results:**

**‘**Keitt’ consistently outperformed ‘Yess!’ in yield efficiency, especially under higher planting density. This advantage was associated with differences in leaf physiological traits (higher stomatal conductance, chlorophyll content and stomatal density) and more dynamic NSC reserve turnover, particularly in trunk tissues. In contrast, ‘Yess!’ maintained larger NSC pools but mobilized reserves less extensively. NSC reserve dynamics aligned with cultivar-specific patterns in carbon allocation and reproductive output.

**Conclusions:**

These findings demonstrate that genotypic variation in NSC regulation is associated with variation in productivity in mango. Higher productivity in ‘Keitt’ was associated with greater seasonal NSC turnover, suggesting stronger source–sink coupling under intensive orchard systems. By integrating measurements of leaf function and NSC reserve mobilization, this study highlights coordinated traits associated with productivity differences among mango cultivars and provides insights relevant to cultivar selection and orchard design.

## INTRODUCTION

Fruit yield in trees is strongly influenced by the availability and partitioning of non-structural carbohydrates (NSCs), particularly their allocation to reproductive structures such as flowers and fruit (Rossouw *et al.*, 2024*b*). NSC supply arises from both concurrent photosynthetic activity and the mobilization of stored reserves that buffer temporal imbalances between carbon assimilation and demand. Carbon partitioning is governed by the balance between source and sink strength, with the biomass ratio of canopy leaf area to crop load acting as a key determinant of whether reserve mobilization is required to sustain fruit development (Sonnewald and Fernie, 2018; Pawar and Rana, 2019).

Leaf-level carbon assimilation, influenced by stomatal conductance and leaf photosynthetic capacity, plays a central role in supporting reproductive development (Flexas and Medrano, 2002; Lu *et al.*, 2012). Stomatal conductance determines the rate of CO_2_ diffusion into leaves, influencing photosynthetic flux, while stomatal density and size affect gas exchange capacity and intrinsic water-use efficiency (Franks and Farquhar, 2007). Chlorophyll content contributes to light capture and can provide an indirect indication of photosynthetic capacity, particularly under conditions where pigment levels reflect nitrogen investment in photosynthetic proteins such as Rubisco, but it does not directly limit assimilation rate (Evans, 1989; Croft *et al.*, 2017). Variability in these traits among genotypes and in response to environment can influence source strength and thus carbon availability for fruit development. However, stomatal behaviour is highly dynamic, responding to microclimatic conditions and phenological stage, which complicates the interpretation of instantaneous gas exchange measurements (Buckley and Mott, 2013; McAusland *et al.*, 2021).

The stable carbon isotope composition (δ^13^C) of plant tissues integrates the relative balance between stomatal conductance and photosynthetic rate over time, and may offer insight into longer-term carbon dynamics (Farquhar *et al.*, 1989; Cernusak *et al.*, 2013). In fruiting species, δ^13^C in mature fruit can reflect canopy photosynthetic performance during fruit development (Gaudillère *et al.*, 2002; Bchir *et al.*, 2016). Similarly, leaf δ^13^C can provide insight into cultivar-specific differences in photosynthetic capacity and stomatal regulation. While δ^13^C has been widely studied in non-climacteric species, its potential application in mango to assess cultivar-specific carbon assimilation and NSC dynamics remains largely unexplored.

NSCs, primarily starch and soluble sugars, are central to tissue growth, plant energy balance, osmotic regulation, metabolic signalling and buffering of environmental or developmental stresses (Smith and Stitt, 2007; Nawaz *et al.*, 2025). Starch functions as an osmotically inert, long-term storage form mainly located in roots and woody tissues like the trunk and branches, while soluble sugars derived from either photosynthesis or starch reserve hydrolysis are transported to sinks via the phloem (Braun, 2022; Landhäusser and Adams, 2024). These dynamics are especially critical during reproductive development, when carbon demand may exceed concurrent photosynthesis under higher crop loads.

Despite their importance, relatively few studies have examined how genotype and tree structure in fruiting species interact to shape NSC dynamics and source–sink relationships over seasonal cycles. In orchard systems, increased planting density is a widely adopted strategy for intensification; however, it may alter light interception and distribution, canopy architecture and internal carbon allocation (Menzel and Le Lagadec, 2017; Haque and Sakimin, 2022). Genotypic differences in vegetative–reproductive balance influence cultivar suitability to such systems (Ibell *et al.*, 2024), yet the underlying physiological mechanisms remain poorly understood. To date, no studies in mango have integrated assessments of leaf functionality, including gas exchange capacity, with NSC dynamics across multiple storage tissues and phenological stages. Such integrative, seasonally resolved assessments remain rare even among other fruit tree species. This type of holistic, temporally resolved approach is essential to understanding how cultivar-specific traits are associated with carbon acquisition, reserve allocation and yield performance under different orchard configurations. This study therefore addresses an important knowledge gap by examining cultivar differences in leaf physiological function and NSC reserve mobilization within a unified source–sink framework. The resulting insights improve understanding of carbon-use and yield efficiencies in intensified orchard systems and may help inform cultivar selection.

This study examined cultivar- and planting density-related differences in leaf function and NSC reserve dynamics in mango, with the aim of identifying physiological mechanisms associated with variation in reproductive performance. Ten-year-old trees of two cultivars differing in yield potential, ‘Keitt’ (higher-yielding) and ‘Yess!’ (lower-yielding), were compared under lower and higher planting densities across two growing seasons. Specifically, we (1) measured stomatal conductance, stomatal morphology, chlorophyll content and δ^13^C as indicators of leaf source function; (2) quantified seasonal changes in NSC concentrations in roots and trunks; and (3) evaluated fruit yield and composition in relation to carbon economy. By integrating these traits over time and across tissues, this study provides the first seasonally resolved framework connecting carbon assimilation, reserve turnover and reproductive output in mango, offering new insight into genotypic adaptation to orchard intensification.

## MATERIALS AND METHODS

### Experimental design

This study was conducted over two consecutive growing seasons (2023–24 and 2024–25) at the Queensland Department of Primary Industries’ Walkamin Research Facility (17°06′S, 145°25′E). Two mango cultivars were evaluated: ‘Yess!’ (also known as ‘NMBP-1243’; an early-maturing hybrid of ‘Irwin’ × ‘Kensington Pride’) and ‘Keitt’ (late-maturing). Each was grown under two contrasting planting densities: low (208 trees ha^−1^; 8 × 6 m spacing) (Supplementary Data Fig. S1) and high (1250 trees ha^−1^; 4 × 2 m spacing) (Supplementary Data Fig. S2). All trees were grafted onto ‘Kensington Pride’ rootstock and planted in December 2013.

Trees were conventionally trained and managed according to standard Australian commercial orchard practices, including fertilization, irrigation and canopy management (Meurant *et al.*, 1999; Ibell *et al.*, 2024). Pruning was conducted within 1 month after harvest each year for both cultivars. Six replicate, free-standing trees per cultivar × density combination were selected from the interior of treatment blocks within a larger, long-term split-split-plot planting system trial (Mahmud *et al.*, 2023; Ibell *et al.*, 2024). A single tree located at the centre of each cultivar sub-plot was used as a replicate for each cultivar and planting density combination. The same individual trees were used across both growing seasons. Tree rows were aligned in a north–south orientation.

### Tree level fruit productivity and composition

Fruit were harvested at physiological maturity (BBCH growth stage 800), based on the BBCH (*Biologische Bundesanstalt, Bundessortenamt und Chemische Industrie*) scale, a standardized system for describing phenological development (Hernández Delgado *et al.*, 2011). Harvest data were collected at the end of the 2022–23 season (prior to the study period) and at the conclusion of the two subsequent growing seasons (2023–24 and 2024–25). Maturity was determined based on external skin colour change and when the fruit flesh dry matter concentration reached at least 12 % (de Freitas *et al.*, 2022). Harvest dates for ‘Yess!’ were 22 December 2022, 2 January 2024 and 17 December 2024, and for ‘Keitt’ they were 18 January 2023, 31 January 2024 and 3 February 2025. For each tree, total fruit number and weight were recorded. Canopy volume was measured annually in November using a spherical volume approximation based on canopy height and width (Mahmud *et al.*, 2023), and yield efficiency was calculated as the ratio of total fruit yield to canopy volume (kg fruit m^−3^ canopy).

In the 2023–24 and 2024–25 seasons, 50 representative fruit per tree were sampled at harvest to determine dry matter content, with 25 fruits randomly selected from both the eastern and western sides of each tree. For each fruit, flesh was extracted using a 6 cm × 2 cm stainless steel corer, combining tissue from the centre of both the blushed and non-blushed sides where possible. Samples were weighed, dried at 60 °C to constant weight, and reweighed to calculate dry matter percentage (Simmons *et al.*, 1998). Dry yield was calculated by multiplying the fresh yield by the average dry matter percentage per tree. Dry yield efficiency was calculated by multiplying fresh yield efficiency by the corresponding dry matter percentage, providing an estimate of dry fruit production per unit canopy volume. This calculation follows the same principle used to express canopy or yield efficiency (yield per canopy volume) (Rosati *et al.*, 2017; Toft *et al.*, 2019) and was derived to allow comparison of productivity on a dry-matter basis.

An additional subsample, pooled from 20 fruits per tree and taken from the blushed side, was frozen at −80 °C, ground under liquid nitrogen using an A11 basic analytical mill (IKA, Selangor, Malaysia) and freeze-dried using a BK-FD18 freeze dryer (Biobase, Shandong, China), and a subsample was homogenized to a fine powder with a TissueLyser II bead mill (Qiagen, Clayton, Australia). These samples were then used for δ^13^C and NSC analyses.

In 2023–24, δ^13^C analysis was performed at the Australian National University’s Stable Isotope Laboratory (Canberra, ACT) using an EA-1110 elemental analyser (Carlo Erba, Milan, Italy) coupled to an isotope ratio mass spectrometer (Isoprime, Micromass, Manchester, UK). In 2024–25, samples were analysed at James Cook University’s Advanced Analytical Centre (Cairns, QLD) using a Flash EA IsoLink™ CN IRMS (Thermo Fisher Scientific, Waltham, MA, USA). Isotope ratios (^13^C/^12^C) were expressed relative to the Vienna Pee Dee Belemnite (VPDB) standard (Farquhar *et al.*, 1989).

Starch, sucrose, glucose and fructose concentrations in the finely ground fruit samples were quantified enzymatically using standard kits (K-TSTA and K-SUFRG; Megazyme International, Bray, Ireland) (Smith and Holzapfel, 2009; Rossouw *et al.*, 2024*a*). Total soluble sugar (cumulative totals of sucrose, glucose and fructose) and total NSC (total sugars plus starch) concentrations were calculated accordingly. Tree-level NSC content in fruit was estimated by multiplying fruit dry yield per tree by the corresponding concentration values obtained from compositional analyses.

### Leaf functionality

Leaf stomatal conductance and chlorophyll content were measured monthly from May to December using ten tagged leaves per tree (five per east and west canopy side). Young leaves were tagged at the shoot apex upon maturation of the latest vegetative flush in May of each season. Stomatal conductance was measured with an LI-600 porometer (LI-COR, Lincoln, NE, USA) under ambient light conditions, and chlorophyll content with a MC-100 chlorophyll meter (Apogee Instruments, Logan, UT, USA), an established non-destructive proxy for leaf chlorophyll concentration rather than a direct quantitative measure (Richardson *et al.*, 2002). Ambient photosynthetic photon flux density recorded by the porometer at the time of each measurement ranged from 8 to 2884 µmol m^−2^ s^−1^ across the seasons (mean ± s.d. = 744 ± 670 µmol m^−2^ s^−1^), reflecting natural within-canopy and temporal variation under field conditions across the study period. For each leaf, three chlorophyll readings were taken at three positions across the lamina and averaged to obtain a single value per leaf. Measurements were conducted between 1030 and 1230 h and grouped according to four phenological stages, based on the BBCH scale: vegetative growth (BBCH 319–321; May), flower formation (BBCH 510–514; June and July), panicle growth (BBCH 515–629; August and September) and fruit development (BBCH 701–709; October to December).

Stomatal density and size were assessed in the 2023–24 season using abaxial leaf imprints collected in November 2023 (BBCH 703–705) from each of the same tagged leaves used for stomatal conductance and chlorophyll measurements. Clear nail polish was applied to the abaxial surface in the lower left quadrant of the lamina, approximately halfway between the midrib and the leaf margin and at least 5 mm from both, to avoid uneven stomatal distributions that may occur near major veins, the midrib and margins (Kardel *et al.*, 2010; Shi *et al.*, 2023). Impressions were taken ∼5 cm above the basal tip of the leaf. The dried impression was peeled off using clear adhesive tape and mounted onto microscope slides. Imprints were examined under a light microscope at 400× magnification (Leitz Dialux 20 EB, Leica Microsystems, Wetzlar, Germany) (Grant and Vatnick, 2004). Stomatal density was determined from three 1-mm^2^ areas per imprint. Stomatal size was estimated by measuring the length and width of nine representative stomata per area, defined as the maximum guard cell complex length and width, with area calculated as an ellipse (Zhang *et al.*, 2023).

Leaf discs were collected during fruit development (BBCH 703–705) on 13 November 2023 and 4 November 2024 by sampling five 6-mm discs per tagged leaf (50 discs per tree). Discs were collected between 0930 and 1100 h, stored on ice to minimize respiration after sampling, oven-dried at 60 °C, and ground to a fine powder using a bead mill. NSC concentrations were quantified enzymatically, and leaf δ^13^C was analysed using the same methods as for fruit tissue.

### Carbohydrate reserves

Root and trunk tissues were sampled for NSC analysis at five phenological stages in each season: initial vegetative flush maturity following pruning (BBCH 319; 9 March 2023 and 6 March 2024 for ‘Yess!’, and 24 March 2023 and 4 April 2025 for ‘Keitt’), reproductive budburst (BBCH 513; 20 June 2023 and 27 June 2023 for ‘Yess!’, and 7 July 2023 and 9 July 2024 for ‘Keitt’), full flowering (BBCH 615; 21 August 2023 and 21 August 2024 for ‘Yess!’, and 1 September 2023 and 4 September 2024 for ‘Keitt’), early fruit growth post-set (BBCH 701; 30 October 2023 and 9 October 2024 for both cultivars), and fruit physiological maturity (BBCH 800; 2 January and 16 December 2024 for ‘Yess!’, and 31 January 2024 and 1 February 2025 for ‘Keitt’).

Roots (3–5 mm diameter) were sampled in an alternating pattern around each tree (north, east, south and west), within 20 cm of the soil surface. These medium-diameter roots were excavated, washed to remove soil, and trimmed to exclude dead or decomposing tissue. Samples were transported to the laboratory on ice to minimize respiration, then oven-dried at 60 °C to constant weight (Smith and Holzapfel, 2009; Rossouw *et al.*, 2024*a*). Dried roots were ground using a hammer mill (DFH 48, Culatti, Glen Creston, Stanmore, UK) and homogenized to a fine powder using a bead mill.

Trunk wood was sampled by drilling to a depth of ∼5 cm at 5–15 cm above the graft union using a 4.8-mm drill bit (Smith and Holzapfel, 2009), with two cores taken per tree from orientations matching root sampling. Samples were kept on ice during transport to the laboratory, then oven-dried and ground to a fine powder using a bead mill. NSC concentrations were determined enzymatically, consistent with leaf and fruit analyses.

### Statistical analysis

Continuous variables measured at a single phenological stage in a season were analysed using ANOVA. These included fruit productivity, dry matter, stomatal density and size, leaf NSC and leaf and fruit δ^13^C. For stomatal conductance each phenological stage within a season was also analysed separately and if multiple assessments were made within a stage, the mean tree data were analysed. This stage-specific approach allows treatment differences to be interpreted under comparable environmental conditions, avoiding confounding effects arising from day-to-day environmental variation in factors such as light intensity, leaf temperature and vapour pressure deficit (Medrano *et al.*, 2002; Buckley, 2005; Urban *et al.*, 2017). The fixed effect in the ANOVA comprised a single factor representing combinations of cultivar and planting density (treatment) and a factor representing replicate was fitted as the random term. Comparisons were also made to investigate the overall main effects of cultivar and planting density. A log_10_ transformation was required for the analysis of stomatal conductance at the fruit growth stage to satisfy the homogeneity of variance assumption. The number of fruits per tree was assumed to follow a negative binomial distribution and was analysed using a Poisson generalized linear model with a natural log link function. Terms fitted in the model include replicate block and a factor representing the four treatments.

Differences between phenological stages within each season were investigated by repeated measures linear mixed model for leaf chlorophyll content and root and trunk starch, total sugars and total NSC concentrations. The fixed effects included treatment nested within phenological stage, and terms for replicate block and plot were fitted as the random effects. Data from each season were analysed separately. A log_10_ transformation was required for the analysis of root starch concentration and root total NSC in 2024–25 to satisfy the homogeneity of variance assumption. For all ANOVA and linear mixed models, the assumptions were assessed by investigating residual diagnostic plots and transformations applied when required. All significance testing was performed at the 0.05 level, and where a significant effect was detected pairwise comparisons were made using the Bonferroni correction.

To assess functional linkages in leaf functionality, NSC reserve mobilization and reproductive output, Spearman rank correlation analyses were performed on leaf traits (including stomatal conductance, chlorophyll content, NSC status and δ^13^C, all measured during the fruit growth period), root and trunk NSC depletion metrics during reproductive development (post-flowering and during fruit growth), and yield and yield efficiency across the two seasons. As stomatal morphology was assessed only in 2024–25, the correlation analysis incorporating size and density was conducted for that season only. All analyses were performed in Genstat 24th edition statistical software (VSN International, Hemel Hampstead, UK).

## RESULTS

### Fruit productivity and composition (2022–23 to 2024–25)

Mean fruit number and yield per tree were greater under low compared with high planting density for all seasons (Table 1). At high density, ‘Keitt’ produced substantially higher yields than ‘Yess!’, with, on average, approximately twice as many fruits and total yield per tree across the three harvests. Mean fruit weight varied by season and cultivar but showed no consistent trend (Table 1).

**Table 1.** Fruit yield, fruit number, average fruit weight, canopy volume and yield efficiency for four cultivar × planting density combinations across three growing seasons (2022–23 to 2024–25).

Variable	‘Yess!’		‘Keitt’		(s.e.)	*P*-value
	Low density	High density	Low density	High density		
2022–23						
Yield (kg per tree)	82.7 ^b^	26.8 ^c^	132.3 ^a^	64.2 ^b^	(6.5)	<0.001
Fruits per tree	146.9 ^b^	49.6 ^c^	280.1 ^a^	143.2 ^b^		<0.001
(s.e.)	(13.1)	(5.4)	(23.6)	(12.9)		
Fruit weight (g per fruit)	563.8 ^a^	551.3 ^b^	473.4 ^a,b^	449.4 ^b^	(25.3)	0.013
Canopy volume (m^3^ per tree)	28.2 ^a^	6.1 ^c^	17.3 ^b^	5.2 ^c^	(1.1)	<0.001
Yield efficiency (kg m^−3^)	2.9 ^c^	4.4 ^c^	7.9 ^b^	12.5 ^a^	(0.6)	<0.001
2023–24						
Yield (kg per tree)	104.1 ^a^	33.8 d	116.5 ^a^	57.6 ^c^	(4.0)	<0.001
Fruits per tree	310.0 ^a^	101.4 ^c^	356.3 ^a^	155.2 ^b^		<0.001
(s.e.)	(20.1)	(7.8)	(22.8)	(11.0)		
Fruit weight (g per fruit)	336.1	337.0	330.7	375.7	(13.4)	0.108
Canopy volume (m^3^ per tree)	26.6 ^a^	3.4 ^c^	16.2 ^b^	3.1 ^c^	(1.7)	<0.001
Yield efficiency (kg m^−3^)	4.2 ^c^	10.1 ^b^	7.3 ^c^	19.0 ^a^	(0.7)	<0.001
Dry matter (%)	12.20	11.9	11.8	12.2	(0.2)	0.606
Dry yield (kg per tree)	12.7 ^a^	4.0 ^c^	13.8 ^a^	7.0 ^b^	(0.5)	<0.001
Dry yield efficiency (kg m^−3^)	0.5 ^c^	1.2 ^b^	0.9 b^c^	2.3 ^a^	(0.1)	<0.001
Fruit starch content (kg per tree)	3.4 ^b^	1.1 d	4.6 ^a^	2.3 ^c^	(0.2)	<0.001
Fruit sugar content (kg per tree)	4.1 ^a^	1.2 ^b^	3.4 ^a^	1.8 ^b^	(0.2)	<0.001
Fruit NSC content (kg per tree)	7.5 ^a^	2.3 ^c^	8.0 ^a^	4.2 ^b^	(0.3)	<0.001
2024–25						
Yield (kg per tree)	102.3 ^a^	28.5 ^c^	116.8 ^a^	59.8 ^b^	(4.3)	<0.001
Fruits per tree	260.1 ^a^	72.8 ^c^	244.8 ^a^	131.1 ^b^		<0.001
(s.e.)	(20.7)	(6.9)	(19.6)	(11.3)		
Fruit weight (g per fruit)	397.2 ^b^	402.2 a^b^	494.2 ^a^	459.2 a^b^	(22.0)	0.019
Canopy volume (m^3^ per tree)	26.3 ^a^	3.0 ^c^	13.8 ^b^	2.3 ^c^	(1.8)	<0.001
Yield efficiency (kg m^−3^)	4.1 ^b^	9.7 ^b^	9.1 ^b^	26.2 ^a^	(1.3)	<0.001
Dry matter (%)	12.6	12.9	12.8	12.8	(0.2)	0.723
Dry yield (kg per tree)	12.9 ^b^	3.7 d	14.9 ^a^	7.7 ^c^	(0.4)	<0.001
Dry yield efficiency (kg m^−3^)	0.5 ^c^	1.3 ^b^	1.2 ^c^	3.3 ^a^	(0.2)	<0.001
Fruit starch content (kg per tree)	4.2 ^b^	1.2 d	6.7 ^a^	3.3 ^c^	(0.2)	<0.001
Fruit sugar content (kg per tree)	4.1 ^b^	1.2 d	5.2 ^a^	2.7 ^c^	(0.2)	<0.001
Fruit NSC content (kg per tree)	8.2 ^b^	2.4 d	11.9 ^a^	6.0 ^c^	(0.3)	<0.001

Dry matter content, dry yield, dry yield efficiency, and fruit NSC contents (starch, sugars and total NSC per tree) were additionally assessed in the 2023–24 and 2024–25 seasons.

Values are means, standard errors and *P*-values.

Within each row, means without a superscript lowercase letter in common indicate significant differences (*P* < 0.05).

For fruit number, standard errors are reported separately for each cultivar × planting density combination, in line with the statistical analysis.

Mean canopy volume was greater under low density for both cultivars (Table 1). ‘Yess!’ had larger canopy volumes than ‘Keitt’ at low density, whereas canopy volume was similar between cultivars at high density. Mean yield efficiency (kg fruit m^−3^ canopy volume) was greater under high density and consistently higher for ‘Keitt’ compared with ‘Yess!’ (Table 1). Mean fruit dry matter content did not differ significantly among cultivar × density combinations within seasons (Table 1). Consequently, dry yield and dry yield efficiency followed similar patterns to fresh yield and yield efficiency (Table 1). Yield and yield efficiency were significantly negatively correlated across the two seasons assessed (*r* = −0.428; *P* = 0.002; Fig. 1).

**Fig. 1.**
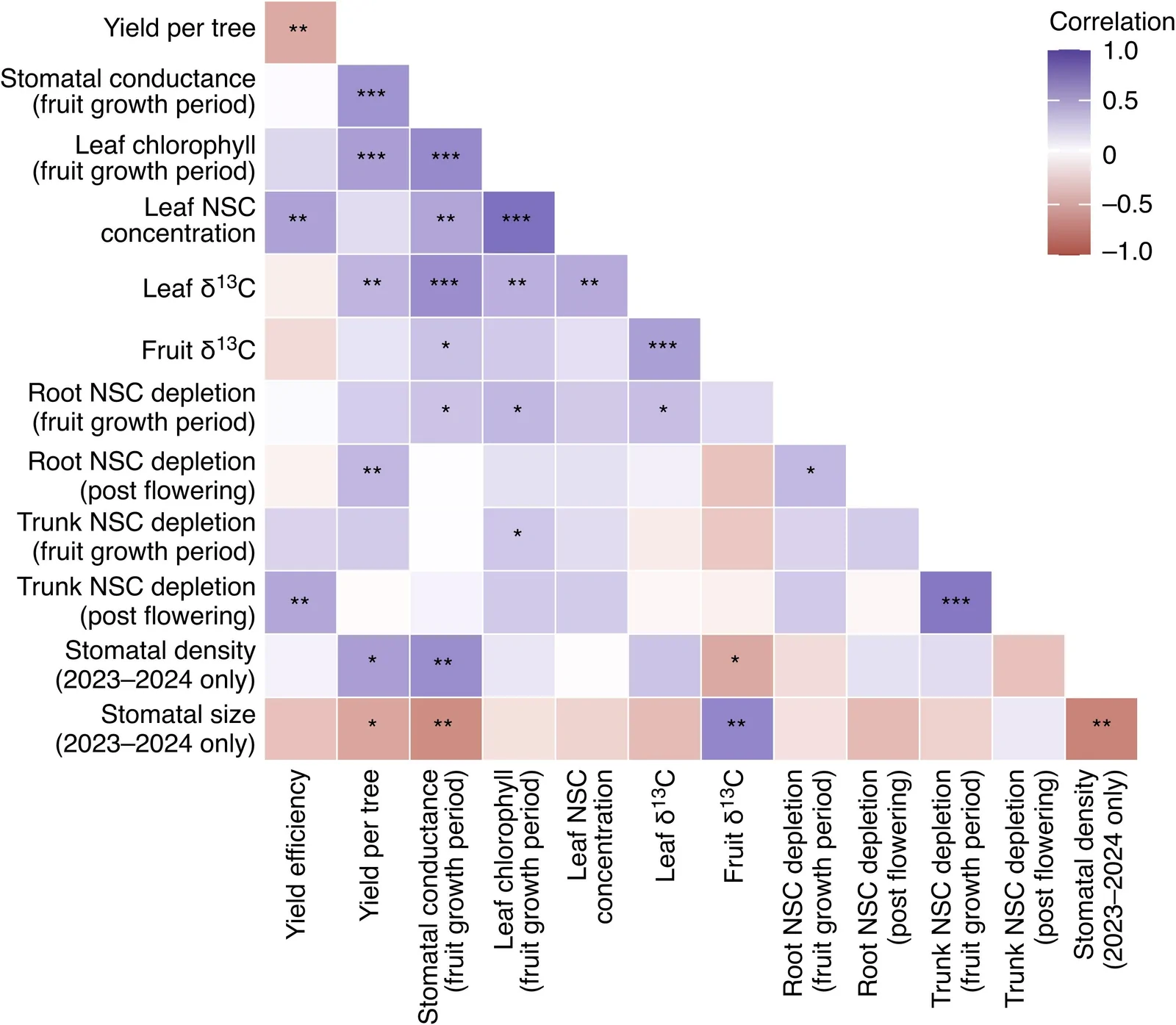
Spearman rank correlations among reproductive performance (yield per tree and yield efficiency), leaf traits [stomatal conductance, chlorophyll content, NSC concentration, stable carbon isotope composition (δ^13^C) and fruit δ^13^C] and root and trunk total NSC depletion during the post-flowering period and fruit growth. Additional leaf traits (stomatal density and size) were assessed only in the 2023–24 season; correlations involving these traits therefore reflect that season only, whereas correlations for all other traits are based on combined data from the 2023–24 and 2024–25 seasons. Colour intensity represents the strength and direction of correlations. as indicated in the key (blue, positive; red, negative). *P < 0.05, **P < 0.01, ***P < 0.001.

Means for fruit total starch, soluble sugar and NSC content per tree were significantly greater under low density for both cultivars (Table 1), with ‘Keitt’ accumulating more fruit starch than ‘Yess!’ at both densities in both seasons assessed. At high density ‘Keitt’ had higher mean sugar content than ‘Yess!’ at both harvests assessed. Within each planting density, total fruit NSC per tree was generally higher in ‘Keitt’, with the difference most pronounced under high density.

### Leaf functionality (2023–24 and 2024–25)

#### Stomatal conductance

Stomatal conductance was analysed among cultivar × planting density combinations at each phenological stage separately to account for short-term variability of this trait (Fig. 2). Across the full dataset, stomatal conductance was not significantly correlated with ambient photosynthetic photon flux density recorded at the time of measurement (*R*^2^ = 0.006; Supplementary Data Fig. S3), indicating that the variation in light exposure during the study was unlikely to confound cultivar or planting density comparisons.

**Fig. 2.**
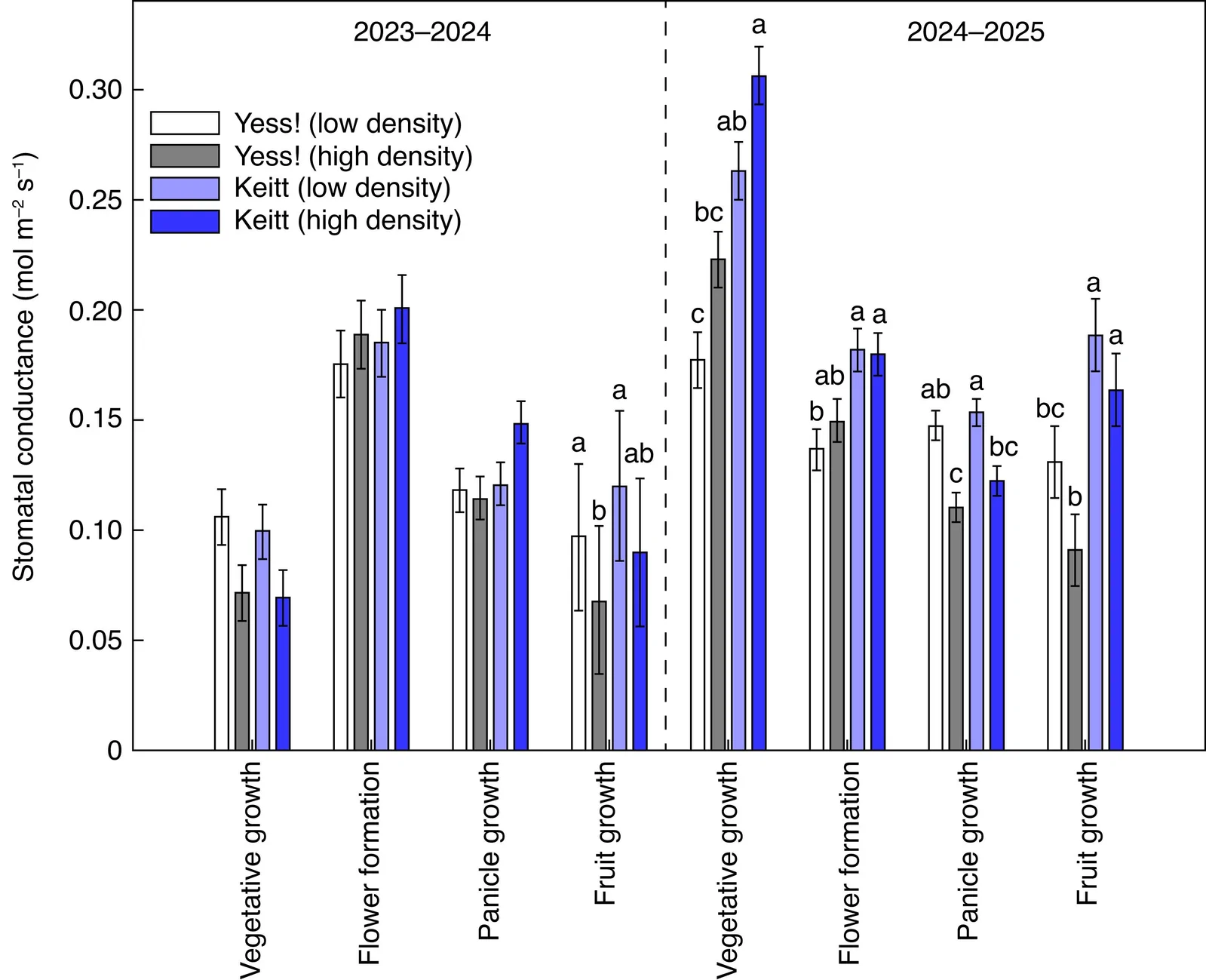
Leaf stomatal conductance for individual growth stages (vegetative growth, flower formation, panicle growth and fruit growth) for four cultivar × planting density combinations during the 2023–24 and 2024–25 seasons. Values are means ± standard error. Where different letters appear, significant differences are present within a growth stage (*P* < 0.05).

In the 2023–24 season, stomatal conductance differed little among cultivar × density combinations within phenological stages, except during fruit growth (Fig. 2). During this stage, ‘Keitt’ generally exhibited higher mean conductance than ‘Yess!’ (cultivar main effect, *P* = 0.005). Low-density trees also showed higher conductance during vegetative growth (*P* = 0.022) and fruit growth (*P* < 0.001) across cultivars. In the 2024–25 season, overall mean stomatal conductance was significantly higher in ‘Keitt’ than in ‘Yess!’ during vegetative growth (*P* < 0.001), fruit formation (*P* = 0.001), and fruit growth (*P* = 0.001). Planting density effects varied by stage, with higher conductance at high density during vegetative growth (*P* = 0.004) but higher conductance at low density during panicle growth (*P* < 0.001).

#### Chlorophyll content

In the 2023–24 season, mean chlorophyll content differed significantly among phenological stage × treatment combinations (*P* < 0.001; Fig. 3). From flower formation, mean chlorophyll levels were generally higher in ‘Keitt’ within a planting density, although these differences were not always statistically significant. The contrast among cultivar × density combinations was most pronounced during panicle growth, when ‘Keitt’ under low density showed the highest values and ‘Yess!’ under high density the lowest. In the 2024–25 season, the stage × treatment effect remained significant (*P* < 0.001). ‘Keitt’ consistently showed significantly higher chlorophyll content than ‘Yess!’.

**Fig. 3.**
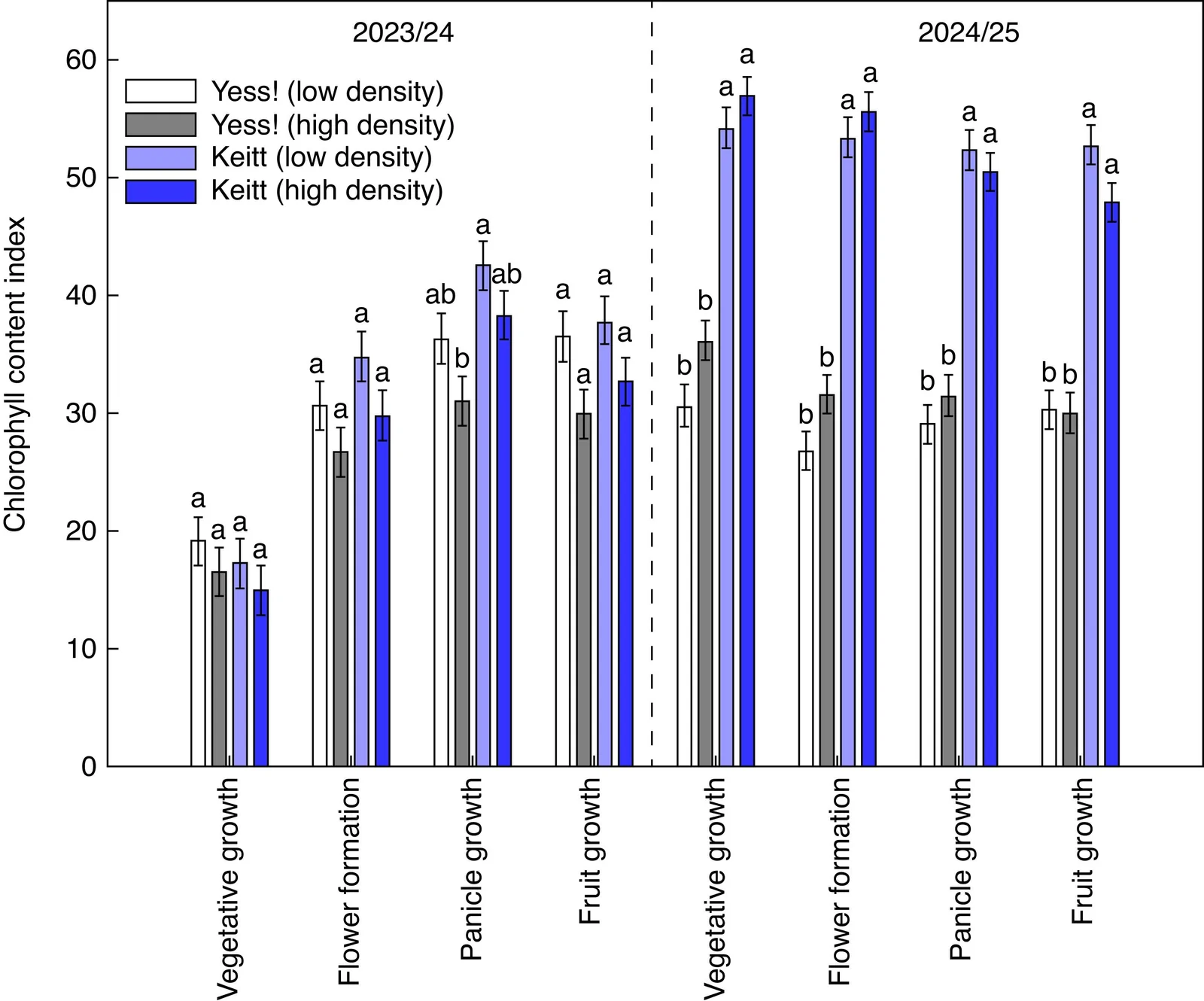
Leaf chlorophyll content index for individual growth stages (vegetative growth, flower formation, panicle growth and fruit growth) for four cultivar × planting density combinations during the 2023–24 and 2024–25 seasons. Values are means ± standard error. Where different letters appear, significant differences are present within a growth stage (*P* < 0.05).

#### Stomatal density and size

Stomatal density and size were only assessed in the 2023–24 season (Fig. 4). Stomatal density was significantly higher for ‘Keitt’ under low planting density compared with ‘Yess!’ (*P* < 0.001). As a main effect, ‘Yess!’ trees exhibited lower stomatal density than ‘Keitt’ trees (*P* < 0.001), with the relative mean increase for ‘Keitt’ being 7 %. For stomatal size, ‘Yess!’ exhibited larger stomata than ‘Keitt’ regardless of planting density (*P* < 0.001), by ∼13 % on average.

**Fig. 4.**
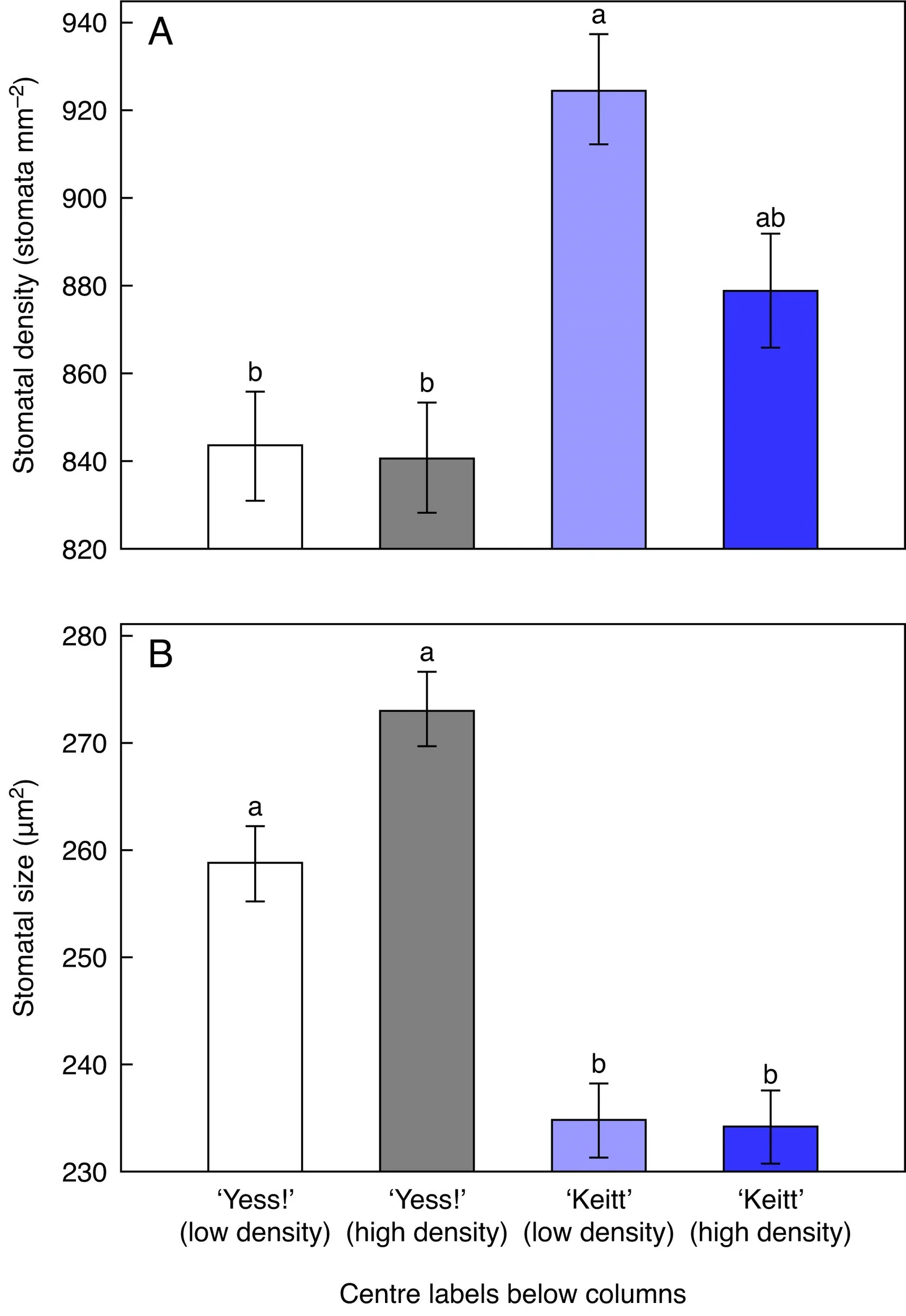
Stomatal density (A) and stomatal size (B) in leaves of ‘Yess!’ and ‘Keitt’ mango trees at low and high planting densities during the 2023–24 season. Values are means ± standard error. Different letters indicate significant differences among cultivar × planting density combinations (*P* < 0.05). Stomatal size refers to the calculated area of the stomatal complex, derived from guard cell length and width.

#### Leaf NSCs

Leaf NSC concentrations were assessed during the fruit growth stage in both seasons (Table 2). In 2023–24, mean leaf starch concentration was significantly higher in ‘Keitt’ than in ‘Yess!’ across both planting densities. While total sugars and total NSC concentrations did not differ significantly among cultivar × planting density combinations, the main cultivar effect indicated higher mean total NSC concentration in ‘Keitt’ than in ‘Yess!’ (*P* = 0.015). In 2024–25, ‘Keitt’ exhibited significantly higher overall mean leaf starch, soluble sugar and total NSC concentrations than ‘Yess!’ when both planting densities were combined (*P* < 0.001).

**Table 2.** Leaf starch, sugars and total NSC concentrations and stable carbon isotope composition (δ^13^C) in leaves and fruit of ‘Yess!’ and ‘Keitt’ mango trees at low and high planting densities during the 2023–24 and 2024–25 growing seasons.

Variable	‘Yess!’		‘Keitt’		s.e.	*P*-value
	Low density	High density	Low density	High density		
2023–24						
Leaf starch concentration (%)	0.41^b^	0.37^b^	0.86 ^a^	1.17^a^	(0.08)	<0.001
Leaf sugars concentration (%)	7.62	7.43	7.59	7.51	(0.22)	0.930
Leaf NSC concentration (%)	8.03	7.80	8.45	8.68	(0.24)	0.076
Leaf carbon isotopes (δ^13^C)	−29.62 ^a,b^	−30.14 ^b^	−28.74 a	−29.67 ^a,b^	(0.23)	0.004
Fruit carbon isotopes (δ^13^C)	−27.27 a	−27.62 ^a,b^	−27.87 ^a,b^	−28.41^b^	(0.20)	0.008
2024–25						
Leaf starch concentration (%)	0.33 ^b^	0.35 ^b^	1.60 ^a,b^	2.42 ^a^	(0.34)	0.001
Leaf sugars concentration (%)	7.28 ^b^	7.39 ^b^	8.61 ^a,b^	9.19 ^a^	(0.33)	0.002
Leaf NSC concentration (%)	7.61 ^b^	7.74 ^b^	10.21 ^a^	11.61 ^a^	(0.40)	<0.001
Leaf carbon isotopes (δ^13^C)	−28.60	−28.80	−28.51	−28.79	(0.15)	0.466
Fruit carbon isotopes (δ^13^C)	−26.64	−27.08	−26.91	−27.08	(0.15)	0.176

Values are means, standard errors and *P*-values.

Within each row, means without a superscript lowercase letter in common indicate significant differences (*P* < 0.05).

#### Carbon isotopes

In the 2023–24 season, ‘Keitt’ exhibited less negative mean δ^13^C values overall than ‘Yess!’ in leaves (*P* = 0.009), while in fruit the opposite was observed (*P* = 0.003) (Table 2). For both leaves and fruit, high-density trees had more negative mean values than those from low density trees (*P* = 0.006 and *P* = 0.040, respectively). In the 2024–25 season, mean δ^13^C values in both leaves and fruit did not differ significantly among cultivar × planting density combinations, or between cultivars.

Several leaf attributes were significantly positively correlated across the two growing seasons (Fig. 1). Stomatal conductance showed relationships with chlorophyll content, leaf δ^13^C (*r* = 0.615 and *r* = 0.586, respectively; *P* < 0.001, both), leaf NSC concentration (*r* = 0.464; *P* = 0.001) and fruit δ^13^C (*r* = 0.315; *P* = 0.029). A more negative or less negative leaf δ^13^C tended to correspond with lower or higher stomatal conductance, respectively. Chlorophyll was also correlated with leaf NSC concentration (*r* = 0.748; *P* < 0.001) and leaf δ^13^C (*r* = 0.402; *P* = 0.005). Leaf NSC concentration was also correlated with leaf δ^13^C (*r* = 0.434; *P* = 0.002), and leaf δ^13^C was correlated with fruit δ^13^C (*r* = 0.495; *P* < 0.001). Stomatal density and size were significantly correlated with stomatal conductance, positively (*r* = 0.594; *P* = 0.002) and negatively (*r* = −0.577; *P* = 0.003), respectively, and showed the opposite pattern with fruit δ^13^C, with density negatively (*r* = −0.440; *P* = 0.031) and size positively (*r* = 0.628; *P* = 0.001) associated with δ^13^C. Stomatal density and size were also significantly negatively related to each other (*r* = −0.636; *P* = 0.001).

### NSC reserves (2023–24 and 2024–25)

NSC concentrations in roots and trunks were assessed across five phenological stages: post-pruning vegetative flushing, panicle emergence (reproductive budburst), full flowering, early fruit growth and harvest, during both seasons (Figs 5 and 6). Statistical comparisons were conducted across cultivar × planting density combinations and phenological stages within each season. However, for visual clarity, post hoc lettering in the figures highlights treatment differences within each stage.

**Fig. 5.**
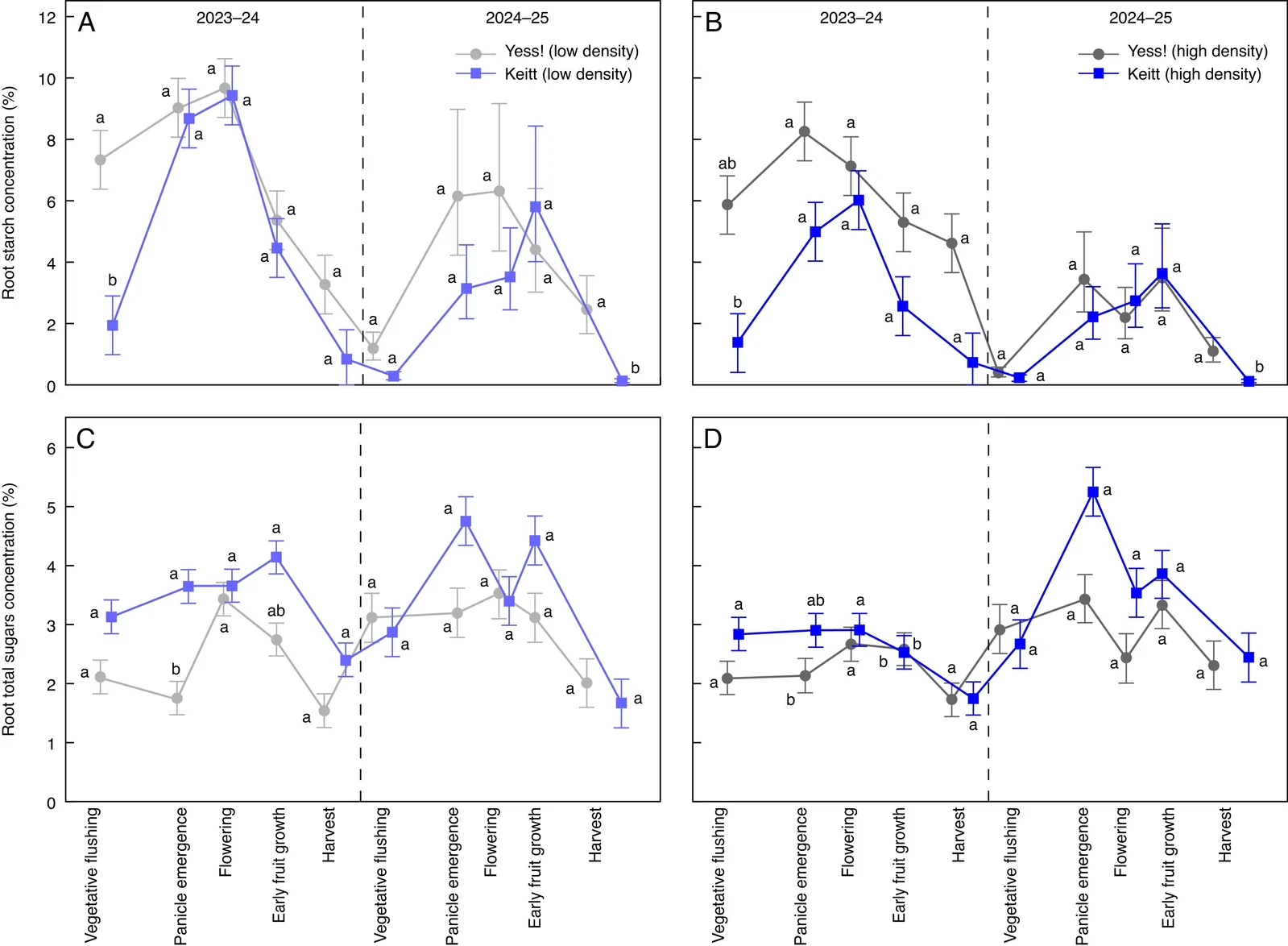
Seasonal changes in root starch (A and B) and total sugar concentrations (C and D) for ‘Keitt’ and ‘Yess!’ mango trees at low and high planting densities during the 2023–24 and 2024–25 seasons. The left-hand panels (A and C) show low-density trees; the right-hand panels (B and D) show high-density trees. Concentrations are presented across five phenological stages: vegetative flush, panicle emergence, flowering, early fruit growth and harvest. Values are means ± standard error. Different lowercase letters indicate statistically significant differences (*P* < 0.05) between cultivar × planting density combinations within each stage and within each season. Statistical comparisons were conducted across treatments and stages within each season; however, for clarity the lettering is adjusted to highlight treatment differences within each stage.

**Fig. 6.**
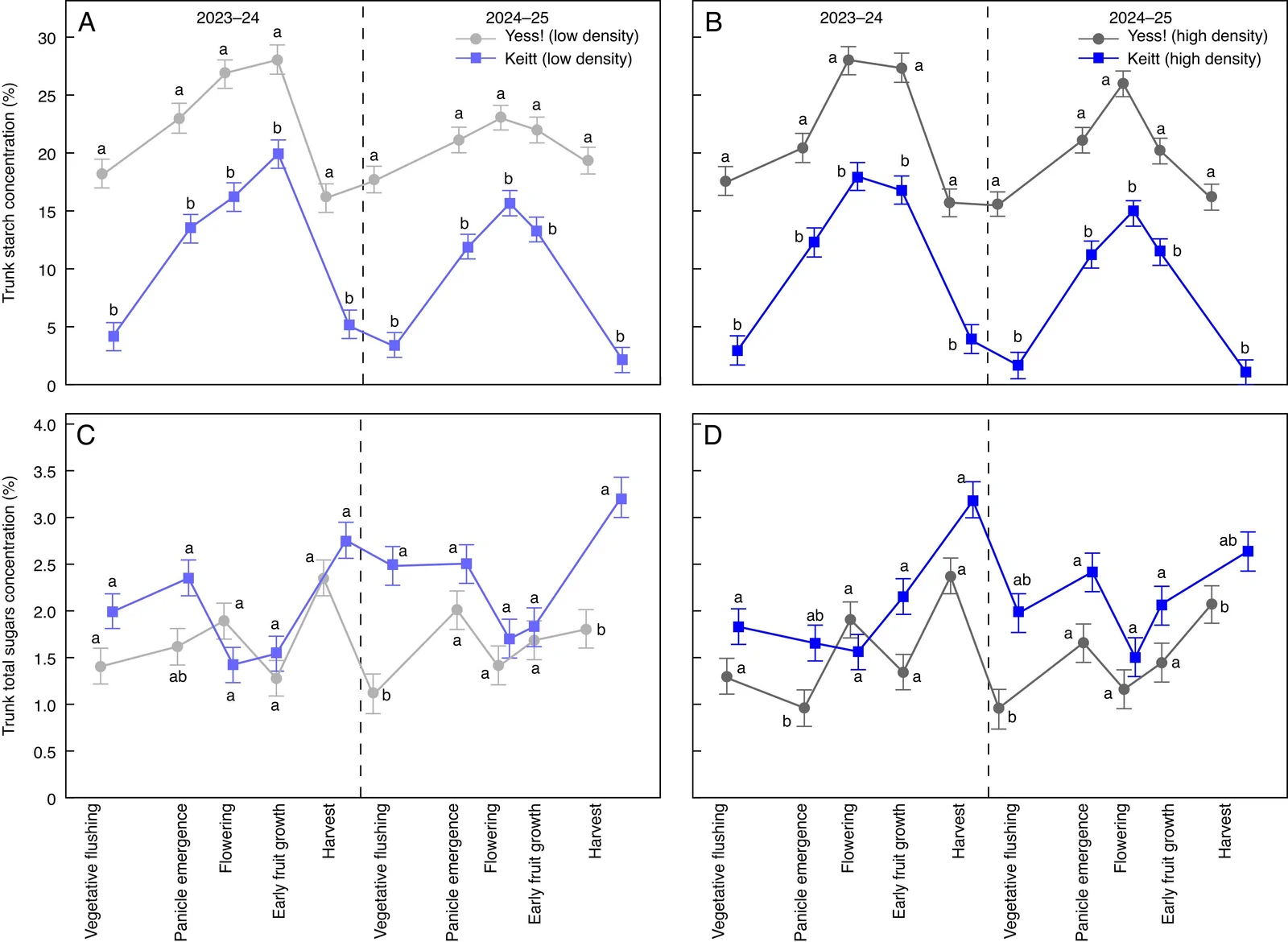
Seasonal changes in trunk starch (A and B) and total sugar concentrations (C and D) for ‘Keitt’ and ‘Yess!’ mango trees at low and high planting densities during the 2023–24 and 2024–25 seasons. The left-hand panels (A and C) show low-density trees; the right-hand panels (B and D) show high-density trees. Concentrations are presented across five phenological stages: vegetative flush, panicle emergence, flowering, early fruit growth and harvest. Values are means ± standard error. Different lowercase letters indicate statistically significant differences (*P* < 0.05) between cultivar × planting density combinations within each stage and within each season. Statistical comparisons were conducted across treatments and stages within each season; however, for clarity the lettering is adjusted to highlight treatment differences within each stage.

#### Roots

Mean root starch concentrations varied significantly across phenological stages and treatments in both seasons (stage × treatment interaction, *P* < 0.001; Fig. 5A, B). Concentrations were generally lower at vegetative flushing, increased towards flowering or early fruit growth, and declined again towards harvest. Peak starch concentrations were generally higher under low planting density. Across both seasons, ‘Keitt’ tended to reach lower starch concentrations at harvest. During vegetative flushing in 2023–24, ‘Keitt’ exhibited lower starch concentrations than ‘Yess!’, whereas concentrations were generally similar between cultivars by flowering across both seasons.

Mean root soluble sugars also showed a significant stage × treatment interaction in both seasons (*P* < 0.001 in 2023–24; *P* = 0.022 in 2024–25; Fig. 5C, D). During vegetative flushing in 2023–24, ‘Keitt’ tended to have higher sugar levels than ‘Yess!’, especially under low density, and this trend persisted towards panicle emergence. By flowering, cultivar differences had largely diminished, and during early fruit growth ‘Keitt’ under low density exhibited the highest sugar concentrations. In 2024–25, ‘Keitt’ generally showed higher sugar concentrations at panicle emergence regardless of planting density. Across both seasons, sugar concentrations declined during fruit growth.

Mean total root NSC concentrations were largely driven by starch and showed a significant stage × treatment interaction in both seasons (*P* < 0.001 in 2023–24; *P* = 0.004 in 2024–25; Supplementary Data Fig. S4). Accordingly, temporal patterns largely mirrored those of starch, with higher concentrations around panicle emergence and flowering, particularly under low planting density, followed by a decline towards harvest. At harvest, ‘Yess!’ generally exhibited greater total NSC concentrations than ‘Keitt’.

#### Trunks

Mean trunk starch concentrations varied significantly across phenological stages and treatments in both seasons (stage × treatment interaction, *P* < 0.001; Fig. 6A, B). ‘Yess!’ had consistently higher starch concentrations than ‘Keitt’, irrespective of planting density. Temporal patterns were similar between seasons, with starch concentrations increasing from the first post-harvest vegetative flush to flowering or early fruit growth, and subsequently declining towards harvest.

Mean trunk soluble sugars also showed a significant stage × treatment interaction in both seasons (*P* < 0.001; Fig. 6C, D). At several stages, ‘Keitt’ tended to exhibit higher sugar concentrations than ‘Yess!’. Across all treatments, sugar concentrations tended to increase during fruit growth, reaching a peak at harvest, when ‘Keitt’ generally exceeded ‘Yess!’.

Mean trunk total NSC concentrations were primarily driven by starch and showed a significant stage × treatment interaction in both seasons (*P* < 0.001; Supplementary Data Fig. S5). Temporal patterns mirrored those of starch, with concentrations increasing from vegetative flushing to peak around flowering before declining during fruit growth. ‘Yess!’ consistently maintained higher total NSC concentrations than ‘Keitt’. However, as with starch, the magnitude of change differed between cultivars, with ‘Keitt’ exhibiting greater increases during early development and more pronounced declines towards harvest than ‘Yess!’. For example, in 2023–24 under low planting density, ‘Keitt’ showed a 3.5-fold increase in NSC concentrations from vegetative flushing to early fruit growth, followed by a 2.7-fold decline by harvest. ‘Yess!’ exhibited only a 1.5-fold increase and a 1.6-fold decline over the same period. A similar pattern was observed in the second season. Total NSC depletion dynamics during both the post-flowering period and fruit growth were not significantly correlated between roots and trunks (*r* = −0.052; *P* = 0.767 and *r* = 0.219; *P* = 0.135, respectively; Fig. 1).

### Productivity in relation to leaf functionality and NSC reserves

Noteworthy relationships between leaf traits and NSC mobilization dynamics and either yield or yield efficiency were identified across the two seasons (Fig. 1). For yield efficiency, significantly positive associations were found with leaf NSC concentration and trunk NSC depletion during the post-flowering period (*r* = 0.476 and *r* = 0.450, respectively; *P* = 0.001, both). For yield per tree, significantly positive associations were found with leaf stomatal conductance and chlorophyll content during fruit growth (*r* = 0.651 and *r* = 0.499, respectively; *P* < 0.001, both), with leaf δ^13^C (*r* = 0.382; *P* = 0.007) and root NSC depletion during the post-flowering period (*r* = 0.434; *P* = 0.002). Leaf stomatal conductance (*r* = 0.298; *P* = 0.040), chlorophyll content (*r* = 0.367; *P* = 0.010) and leaf δ^13^C (*r* = 0.330; *P* = 0.022) were also significantly positively associated with root NSC depletion during fruit growth. Leaf chlorophyll content showed a significant positive association with trunk NSC depletion during fruit growth (*r* = 0.285; *P* = 0.049).

## DISCUSSION

This study integrates leaf functionality, carbohydrate reserve dynamics and yield efficiency within a seasonally resolved framework to understand fruit productivity in mango. This novel approach provides insight into how cultivar-level differences in carbon regulation are associated with yield performance. Fruit productivity was shaped by cultivar-specific traits, with planting density acting as a modifying factor. ‘Keitt’ consistently outperformed ‘Yess!’ in reproductive output.

Although greater yields under low planting density reflected larger canopies, ‘Keitt’ maintained superior yield efficiency, especially under high density. ‘Keitt’ therefore appears more effective at allocating assimilates towards reproductive sinks relative to canopy size, indicating stronger source–sink coordination under spatial constraints (Corelli-Grappadelli and Lakso, 2004; Robinson, 2007). This likely supports key reproductive processes, including flowering, fruit set, retention and growth, and reflects physiological and architectural traits that stabilize reproductive output despite vegetative competition (Capelli *et al.*, 2016; Ryan *et al.*, 2018; Falchi *et al.*, 2020; Jones *et al.*, 2026). The enhanced dry yield metrics for ‘Keitt’ further support cultivar-level differences in carbon partitioning during reproductive development (Génard *et al.*, 2008).

The larger canopies and lower yield efficiency observed in ‘Yess!’ align with its reported vegetative growth bias relative to ‘Keitt’ (Ibell *et al.*, 2024), potentially limiting its suitability for high-density orchard systems. Variation in productivity was primarily driven by fruit number rather than size, a pattern that aligns with findings in other tree crops, such as apple (*Malus domestica*) and citrus (*Citrus* spp.) (Corelli-Grappadelli and Lakso, 2004; Goldschmidt and Sadka, 2021). ‘Keitt’ trees generally accumulated more NSCs in fruit, further indicating greater carbon investment in reproductive sinks (MacNeill *et al.*, 2017; Rossouw *et al.*, 2024*b*).

‘Keitt’ generally exhibited higher leaf stomatal conductance than ‘Yess!’, particularly during the second season and during reproductive development. This pattern is consistent with a greater capacity to sustain gas exchange under strong carbon sink demand and is likely associated with greater photosynthetic potential and more effective assimilate supply to developing fruit (Urban *et al.*, 2004; Lu *et al.*, 2012; Roche, 2015). The influence of planting density on stomatal conductance varied, likely reflecting climatic variation across the study period, including changes in solar exposure, leaf temperature, vapour pressure deficit and water availability as conditions transitioned between the wet and dry season (Medrano *et al.*, 2002; Buckley, 2005; Urban *et al.*, 2017; Supplementary Data Figs S6–S8).

These findings align with previous reports of mango genotypic differences in stomatal behaviour and photosynthetic capacity (Lu *et al.*, 2012), supporting the view that cultivar-specific traits are associated with carbon assimilation dynamics during reproductive development. While stomatal conductance is closely linked to photosynthetic capacity, it does not directly quantify carbon assimilation, as photosynthesis is also influenced by biochemical limitations including Rubisco activity, electron transport capacity and internal CO_2_ diffusion (Farquhar and Sharkey, 1982; Prywes *et al.*, 2023). Nevertheless, a strong relationship between net carbon assimilation and stomatal conductance has been demonstrated in mango (Urban *et al.*, 2002; Lu *et al.*, 2012). The positive associations found between stomatal conductance during fruit growth, leaf NSC concentration and final fruit yield further support a functional link between stomatal regulation, carbon assimilation and fruit production.

‘Keitt’ leaves generally exhibited higher chlorophyll content than ‘Yess!’; this pattern was particularly strong in 2024–25, suggesting a genotypic difference in maintaining leaf pigment levels under varying canopy conditions. Although chlorophyll content does not directly determine photosynthetic capacity, it is closely associated with foliar nitrogen status (Evans, 1989). Photosynthetic capacity is often primarily influenced by stomatal limitations and biochemical factors such as Rubisco activity (Prywes *et al.*, 2023; Scafaro *et al.*, 2023), and therefore chlorophyll content alone should be interpreted cautiously as an indicator of photosynthetic performance. That said, the positive relationships between leaf chlorophyll content and other leaf traits, including stomatal conductance and NSC concentration, suggest that multiple factors associated with photosynthesis were stronger in ‘Keitt’ relative to ‘Yess!’. Furthermore, in mango, leaf nitrogen concentration tends to influence biochemical photosynthetic capacity, showing a positive non-linear relationship with maximum assimilation parameters such as carboxylation, electron transport capacity and triose phosphate utilization (Urban *et al.*, 2003). The higher chlorophyll content observed in ‘Keitt’ during the second season may reflect improved nitrogen uptake or seasonal environmental conditions that favoured nitrogen availability or assimilation (Croft *et al.*, 2014).

‘Keitt’ having higher stomatal density and smaller stomata relative to ‘Yess!’ suggests a genotypic influence on stomatal development (Cai *et al.*, 2024). A denser population of smaller stomata may contribute to greater potential gas exchange capacity, which aligns with ‘Keitt’s higher stomatal conductance. However, the magnitude of cultivar differences was modest, with on average an increase in 7 % for ‘Keitt’. Studies in other species directly linking stomatal density shifts to gas-exchange responses have generally involved much larger changes than those observed here. For example, in sorghum (*Sorghum bicolor*), reductions in stomatal density of 45 and 69 % were associated with 18 and 32 % reductions in stomatal conductance, respectively, with only the stronger reduction increasing stomatal limitation to photosynthesis (Ferguson *et al.*, 2021). Accordingly, the functional significance of the smaller stomatal differences observed remains uncertain. The positive association between stomatal density and conductance suggests a functional link between density and gas exchange, although further research is needed to assess its relationship with net carbon assimilation in mango.

This size–density trade-off whereby ‘Yess!’ exhibits larger but fewer stomata supports a well-recognized balance between maximizing pore number and ensuring sufficient aperture size (Dow *et al.*, 2014). Although larger stomata can support greater conductance per individual pore, a lower density may constrain maximum gas exchange capacity (Drake *et al.*, 2013). In contrast, relative to ‘Yess!’, the stomatal profile of ‘Keitt’ suggests a higher potential maximum conductance (Franks and Beerling, 2009; Harrison *et al.*, 2020). That said, the extent to which these relatively small differences contribute meaningfully to cultivar differences in leaf gas exchange remains unclear.

Although measured at a single time point during fruit growth each season, the elevated leaf NSC status for ‘Keitt’ suggests a greater photosynthetic surplus and short-term carbon storage capacity relative to ‘Yess!’ (MacNeill *et al.*, 2017). Higher starch concentrations in particular suggest greater assimilate storage during the day that could be used at night to sustain sink demand (Smith and Stitt, 2007). The positive relationship between leaf NSC concentration and fruit yield efficiency further supports the view that leaf source function contributes to reproductive output relative to tree size.

The utility of δ^13^C as an integrative indicator of leaf gas exchange was season-dependent. In the first season, it somewhat differentiated between cultivar × planting density combinations in both leaves and fruit, whereas in the second season it did not show clear differences. This inconsistency may reflect differences in environmental and physiological context between seasons. δ^13^C is sensitive to evaporative demand and associated stomatal responses, which influence intercellular CO_2_ concentration and isotopic discrimination (Tieszen, 1991; Seibt *et al.*, 2008). δ^13^C integrates multiple processes beyond stomatal conductance, including mesophyll conductance and biochemical limitations to photosynthesis such as Rubisco carboxylation capacity, which can vary independently of gas exchange patterns (Farquhar and Sharkey, 1982; Seibt *et al.*, 2008). In addition, δ^13^C in fruit tissue may reflect the contribution of stored carbohydrate reserves from woody tissues, whereby remobilized reserves can contribute to fruit carbon pools, decoupling isotopic signatures from concurrent leaf gas exchange (Badeck *et al.*, 2005; Génard *et al.*, 2008).

As a result, these factors can reduce the sensitivity of δ^13^C measurements in detecting moderate treatment differences under certain seasonal conditions. This highlights a key limitation in applying δ^13^C as an integrative proxy for gas exchange in mango and underscores the need for further work to define the environmental and physiological conditions under which it reliably reflects carbon assimilation dynamics. Nevertheless, the generally less negative leaf δ^13^C values in ‘Keitt’ compared with ‘Yess!’ in 2023–24 suggest reduced discrimination against ^13^C during photosynthesis. This pattern typically reflects either lower stomatal conductance or higher photosynthetic activity relative to stomatal aperture (Farquhar *et al.*, 1989; Cernusak *et al.*, 2013). Given the observed stomatal conductance patterns during the corresponding phenological growth stage, together with the positive association between leaf δ^13^C and stomatal conductance, the δ^13^C signal in ‘Keitt’ appears consistent with stronger drawdown of intercellular CO_2_ through higher assimilation. Reduced stomatal opening is therefore unlikely to explain this pattern. Fruit δ^13^C values showed the opposite trend, tending to be more negative in ‘Keitt’ than in ‘Yess!’, which is consistent with sustained stomatal openness or greater cumulative carbon availability during fruit development (Grant *et al.*, 2012). These variable patterns between tissues likely reflect the temporal integration of carbon signals, with leaf δ^13^C representing shorter-term assimilation and fruit δ^13^C reflecting cumulative carbon input during fruit growth (Bchir *et al.*, 2016).

Root NSC dynamics varied modestly between cultivars and across stages, with overall patterns largely shaped by phenology. At some stages across both seasons, ‘Yess!’ exhibited higher root starch concentrations than ‘Keitt’, most clearly at harvest. ‘Keitt’ typically exhibited more pronounced starch depletion during fruit growth, suggesting stronger remobilization of reserves to support reproductive demand (Sala *et al.*, 2012; Pawar and Rana, 2019). However, because crop load and reserve dynamics may influence one another bidirectionally through source–sink feedbacks, the greater reserve depletion observed in ‘Keitt’ may reflect its higher reproductive demand rather than indicating that reserve mobilization alone drives higher productivity (Ryan *et al.*, 2018).

Across both cultivars, root starch concentrations generally peaked at or before flowering and declined thereafter, a pattern consistent with mobilization during increasing carbon demand associated with fruit growth (Rossouw *et al.*, 2024*b*). However, the magnitude and timing of these changes varied with cultivar, planting density and season, highlighting that root reserve turnover is sensitive to both phenological drivers and environmental conditions (Loescher *et al.*, 1990). The positive association between the extent of root total NSC depletion from flowering to harvest and final yield per tree further supports the view that root reserve mobilization and reproductive demand are associated. In contrast, the lack of a similar link between NSC depletion during fruit growth and yield suggests that root reserve mobilization during earlier reproductive stages may interact with eventual tree-level productivity. As all trees were grafted onto a common ‘Kensington Pride’ rootstock, the observed dynamics in root NSC dynamics is most consistent with scion-driven physiological control. Interactions between cultivar traits, canopy size and crop load were likely associated with differences in root reserve turnover, particularly under high sink demand (Rossouw *et al.*, 2017; Tixier *et al.*, 2020).

Cultivar-level differences in reserve accumulation and remobilization were more pronounced in the trunk. Trunk NSC concentrations were generally rebuilt by flowering and subsequently mobilized to support reproductive demand. The positive association between NSC depletion from flowering to harvest and fruit yield efficiency suggests that the extent of trunk reserve mobilization during reproductive development is linked to fruit productivity relative to canopy size. ‘Keitt’ consistently exhibited greater starch rebuilding and depletion dynamics, suggesting a more active mobilization of reserves during reproductive development (Ferguson *et al.*, 2021). In contrast, ‘Yess!’ maintained higher overall starch levels, with comparatively smaller shifts between peaks and troughs, indicative of a more storage-oriented strategy (Loescher *et al.*, 1990). These differences may partly reflect genotypic variation in starch storage capacity, potentially underpinned by anatomical traits such as higher parenchyma density or plastid-rich storage tissues in ‘Yess!’ (Plavcová and Jansen, 2015), and may help explain the cultivar-specific starch storage profiles observed here. However, further investigation is needed to confirm whether such cellular differences exist between mango genotypes.

‘Keitt’ generally maintained higher trunk sugar levels than ‘Yess!’ during reproductive development, suggesting sustained or late-season mobilization of a readily translocatable carbon source (Smith and Stitt, 2007). Overall, these patterns suggest that ‘Keitt’ operates under a more flexible and responsive reserve management strategy, dynamically reallocating stored carbon in line with sink demand. Conversely, ‘Yess!’ appears to follow a more conservative approach, maintaining larger reserve pools but mobilizing them less extensively. These differences may also partly reflect reproductive demand, with reserve depletion responding to crop load rather than solely determining productivity.

The temporal sequence of reserve use, with root depletion tending to precede trunks, supports earlier suggestions that mango trees draw on below-ground reserves early in the season before shifting to above-ground stores (Davie and Stassen, 1997). This sequence suggests that trunk reserves play an important role in sustaining late-stage reproductive development when photosynthetic supply may be insufficient relative to crop load. Moreover, the correlation analyses show that root NSC depletion was associated with total yield per tree, whereas trunk NSC depletion was more associated with yield efficiency. This suggests that root reserve mobilization may be aligned with overall sink strength and crop load (Sonnewald and Fernie, 2018), while trunk reserves may play a more compensatory role in maintaining productivity relative to canopy size, buffering carbon supply during periods of high sink demand (Rossouw *et al.*, 2024*b*).

### Conclusions

This study demonstrates that cultivar-level differences in carbon regulation are associated with variation in mango productivity under contrasting planting densities. Across two seasons, ‘Keitt’ consistently outperformed ‘Yess!’ in yield efficiency, particularly under high-density conditions, reflecting more effective coordination between carbon assimilation, allocation and reserve use. Greater reproductive output was associated with differences in leaf function, including higher stomatal conductance in ‘Keitt’, indicating greater gas exchange capacity and the ability to sustain carbon supply under reproductive demand. These traits were complemented by more dynamic starch reserve turnover in ‘Keitt’, particularly in trunk tissues, consistent with a more responsive mobilization of stored carbon to support fruit development. By integrating leaf physiology and NSC reserve dynamics across developmental stages, this study provides a novel system-level framework linking carbon regulation with fruit productivity in mango. These findings highlight the potential for using physiological traits to guide cultivar selection and support the use of ‘Keitt’-like phenotypes in intensified orchard systems. However, validation across a broader range of cultivars and environments is required to determine the generality of these relationships.

## Supplementary Material

mcag188_Supplementary_Data

## Data Availability

Data are available at the discretion of the corresponding author upon reasonable request.

## SUPPLEMENTARY DATA

Supplementary data are available at *Annals of Botany* online and consist of the following. Figure S1: photograph illustrating the canopy structure of ‘Keitt’ trees grown at low density. Figure S2: photograph illustrating the canopy structure of ‘Keitt’ trees grown at high density. Figure S3: relationship between leaf stomatal conductance and ambient photosynthetic photon flux density. Figure S4: root total NSC dynamics across the two seasons. Figure S5: trunk total NSC dynamics across the two seasons. Figure S6: mean monthly rainfall during the study period. Figure S7: mean maximum and minimum atmospheric temperatures during the study period. Figure S8: mean monthly solar exposure during the study period. Data in Figures S6–S8 are from the onsite weather station, Australian Bureau of Meteorology station ID 31108.

## AI ASSISTANCE

AI was used for minor text improvements and proofreading.
