# Supplementary material for: Coordination of leaf function and carbohydrate reserves is associated with cultivar differences in mango (Mangifera indica) fruit productivity
Source: Ann Bot. 2026 Jun 27;138(5):2014–28. doi: 10.1093/aob/mcag188 (PMC13715004; doi:10.1093/aob/mcag188)
Supplement: Supplementary file 1 [file mcag188_supplementary_data.pdf]

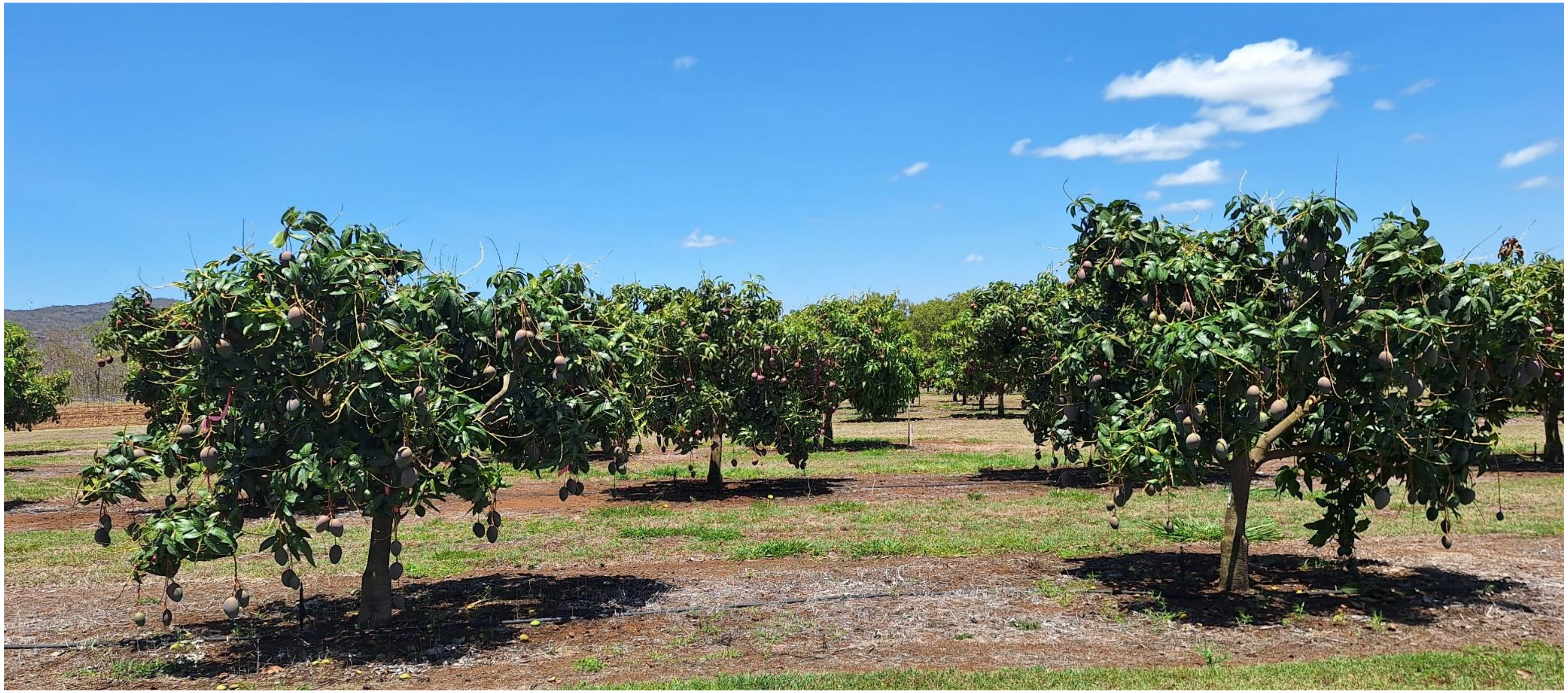

FIG. S1. ‘Keitt’ trees grown at low density (208 trees ha^{-1}; 8 × 6 m spacing) at the Queensland Department of Primary Industries’ Walkamin Research Facility, Far North Queensland, Australia. The photograph was taken during the fruit maturation phase in January 2025.

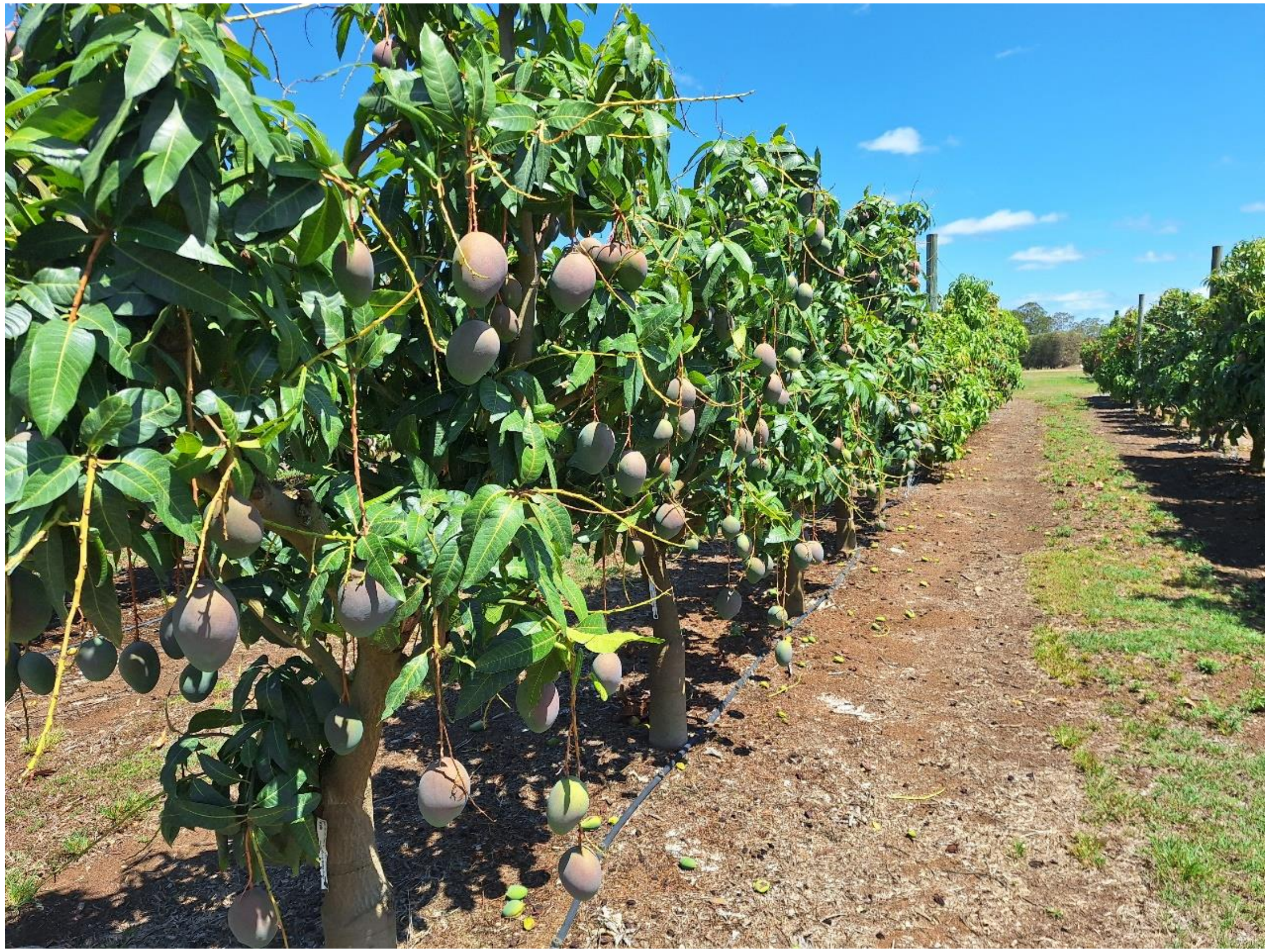

FIG. S2. ‘Keitt’ trees grown at high density (1250 trees ha^{-1}; 4 × 2 m spacing) at the Queensland Department of Primary Industries’ Walkamin Research Facility, Far North Queensland, Australia. The photograph was taken during the fruit maturation phase in January 2025.

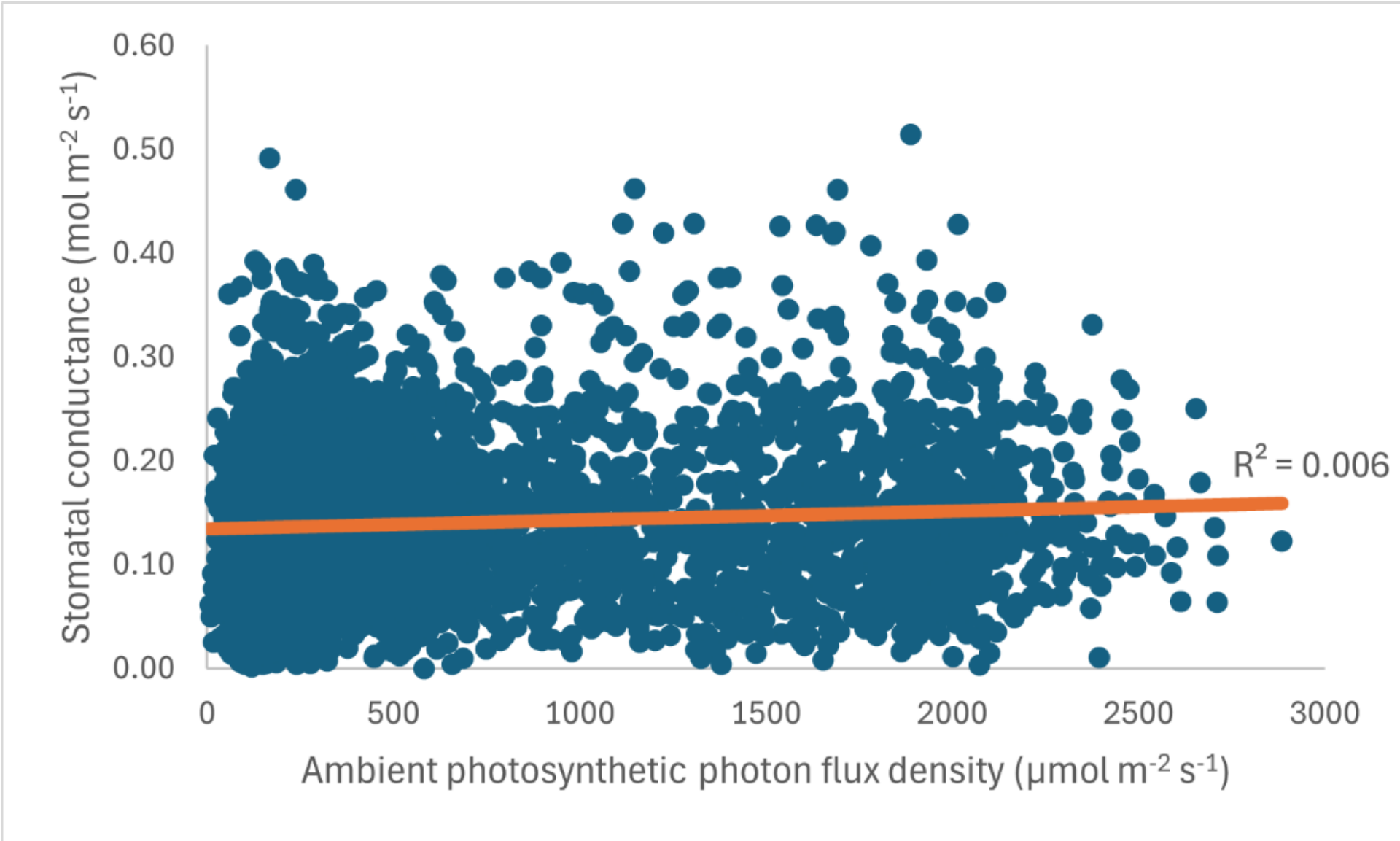

FIG. S3. Relationship between stomatal conductance and ambient photosynthetic photon flux density across all measurements in both seasons (2023-2024 and 2024-2025).

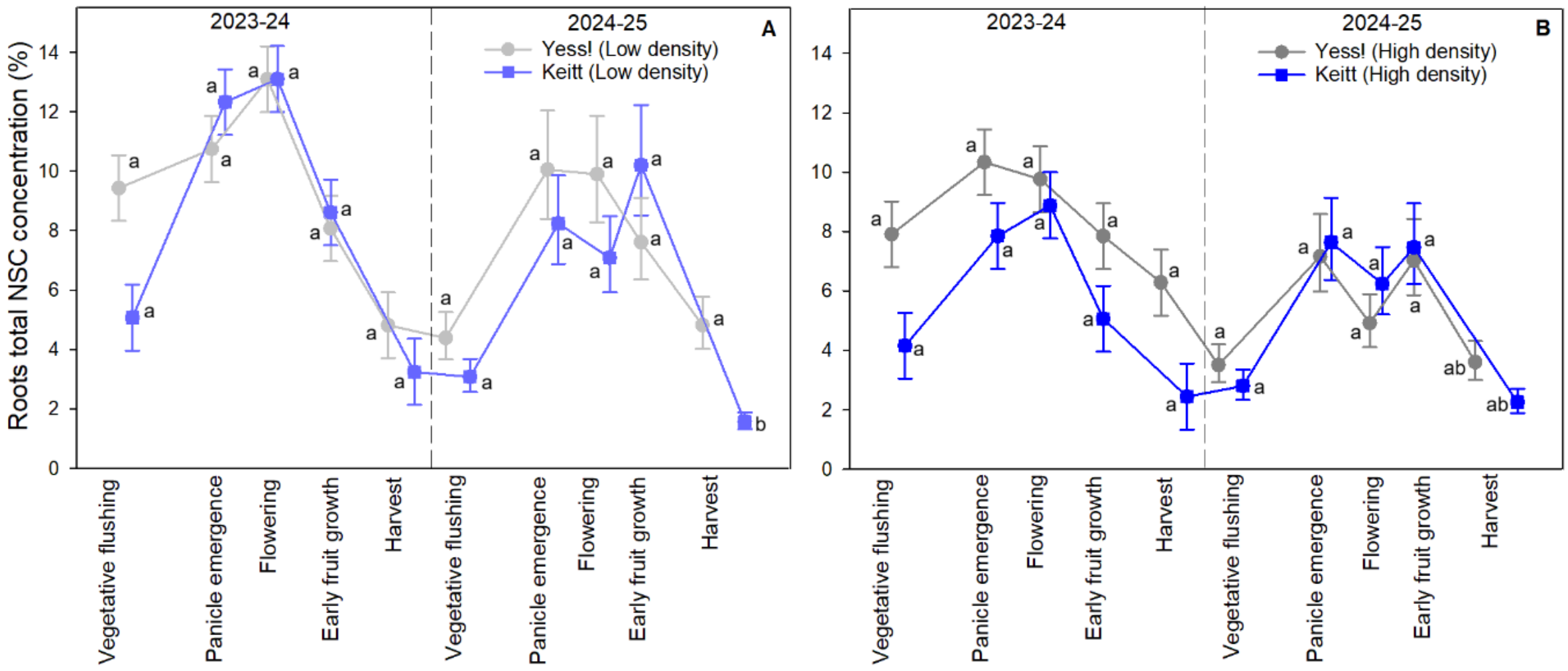

FIG. S4. Seasonal changes in root total non-structural carbohydrate (NSC) concentrations for 'Keitt' and 'Yess!' mango trees at low and high planting densities during the 2023–2024 and 2024–2025 seasons. The left-hand panel (A) show low density trees; the right-hand panel (B) show high density trees. Concentrations are presented across five phenological stages: vegetative flush, panicle emergence, flowering, early fruit growth, and harvest. Values are means ± standard error. Different lowercase letters indicate statistically significant differences (P < 0.05) between cultivar × planting density combinations within each stage and within each season. Statistical comparisons were conducted across treatments and stages within each season, however for clarity, lettering was adjusted to highlight treatment differences within each stage.

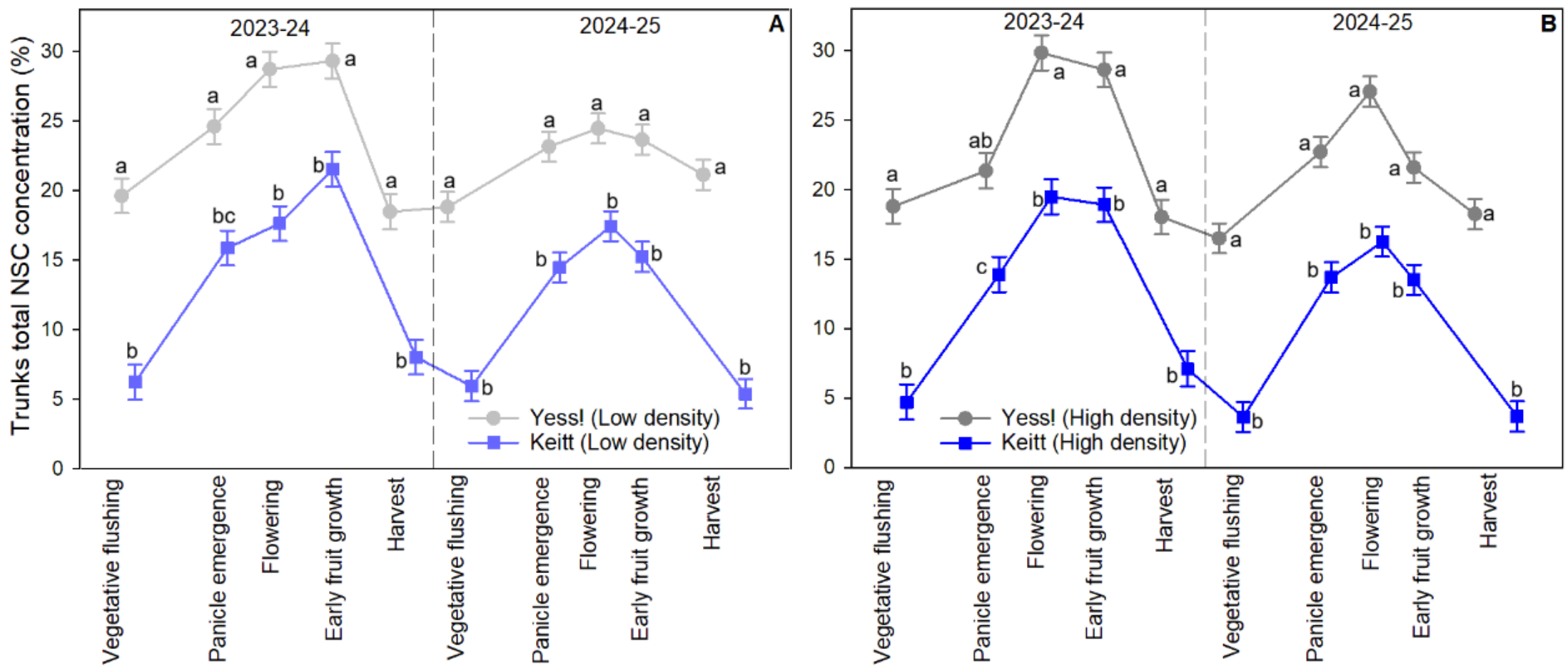

FIG. S5. Seasonal changes in trunk total non-structural carbohydrate (NSC) concentrations for ‘Keitt’ and ‘Yess!’ mango trees at low and high planting densities during the 2023–2024 and 2024–2025 seasons. The left-hand panel (A) show low density trees; the right-hand panel (B) show high density trees. Concentrations are presented across five phenological stages: vegetative flush, panicle emergence, flowering, early fruit growth, and harvest. Values are means ± standard error. Different lowercase letters indicate statistically significant differences ($P < 0.05$) between cultivar × planting density combinations within each stage and within each season. Statistical comparisons were conducted across treatments and stages within each season, however for clarity, lettering was adjusted to highlight treatment differences within each stage.

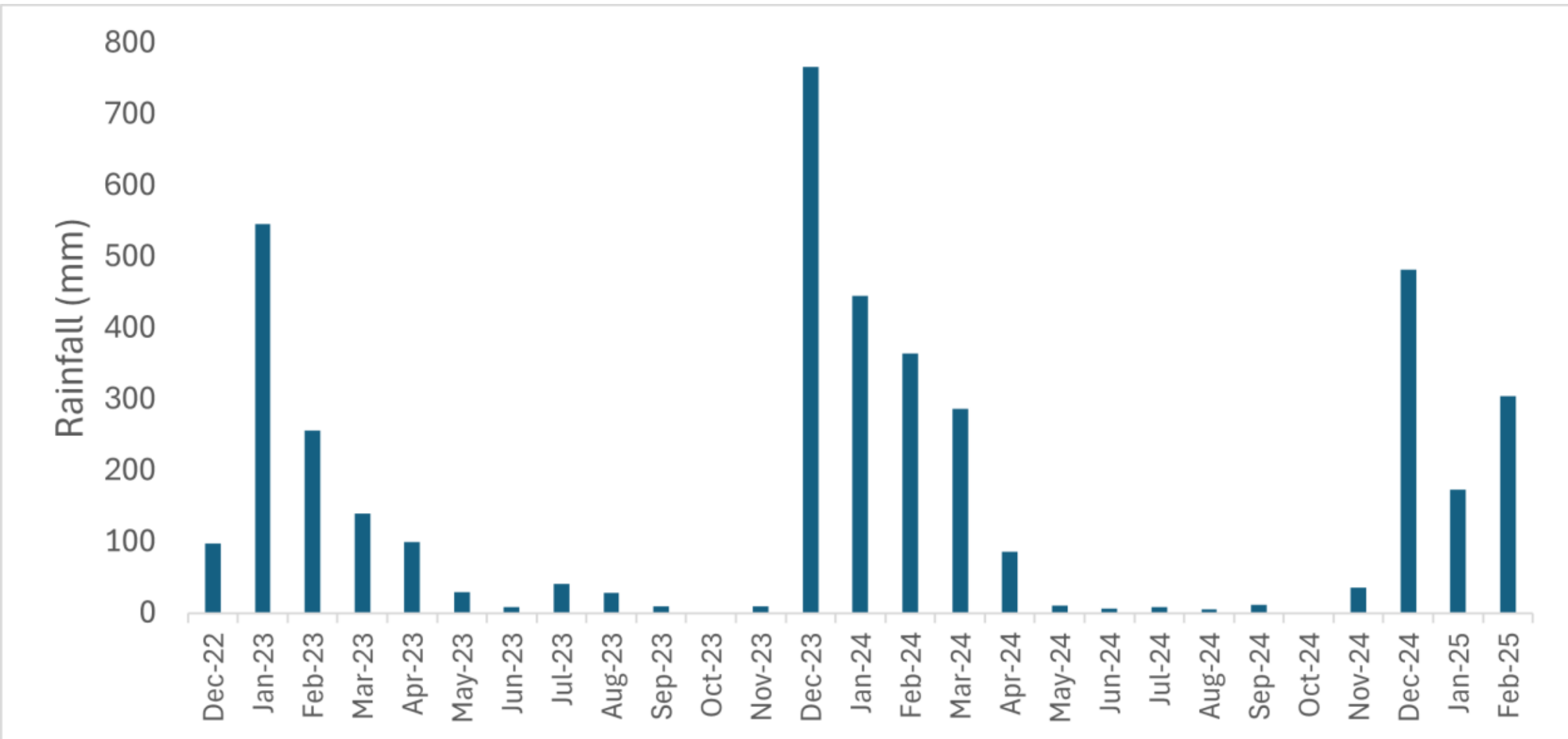

FIG S6. Monthly mean rainfall (mm) recorded at the site across the study period (December 2022 to February 2025) encompassing both growing seasons (2023-2024 and 2024-2025). Data were obtained from the Australian Bureau of Meteorology station at Walkamin Research Station (station ID 31108).

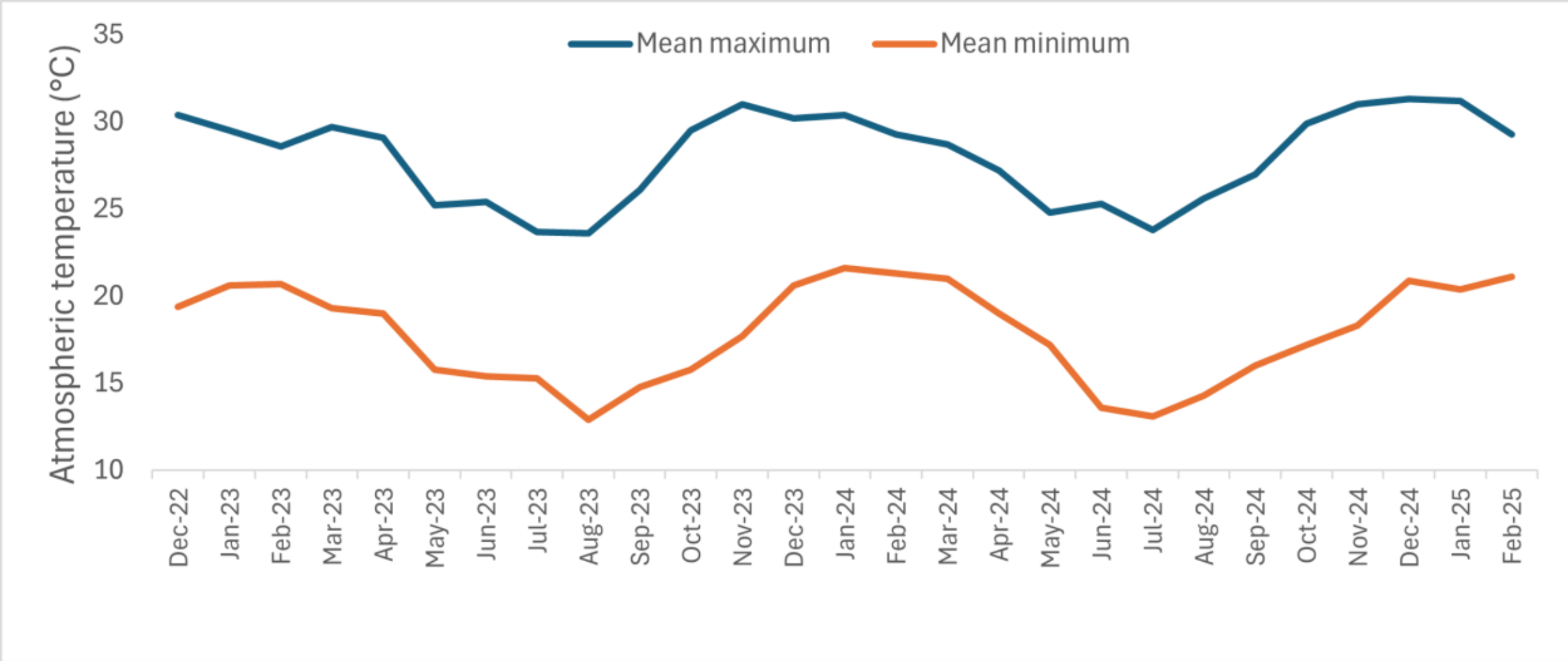

FIG S7. Monthly mean maximum and minimum atmospheric temperatures recorded at the site across the study period (December 2022 to February 2025) encompassing both growing seasons (2023-2024 and 2024-2025). Data were obtained from the Australian Bureau of Meteorology station at Walkamin Research Station (station ID 31108).

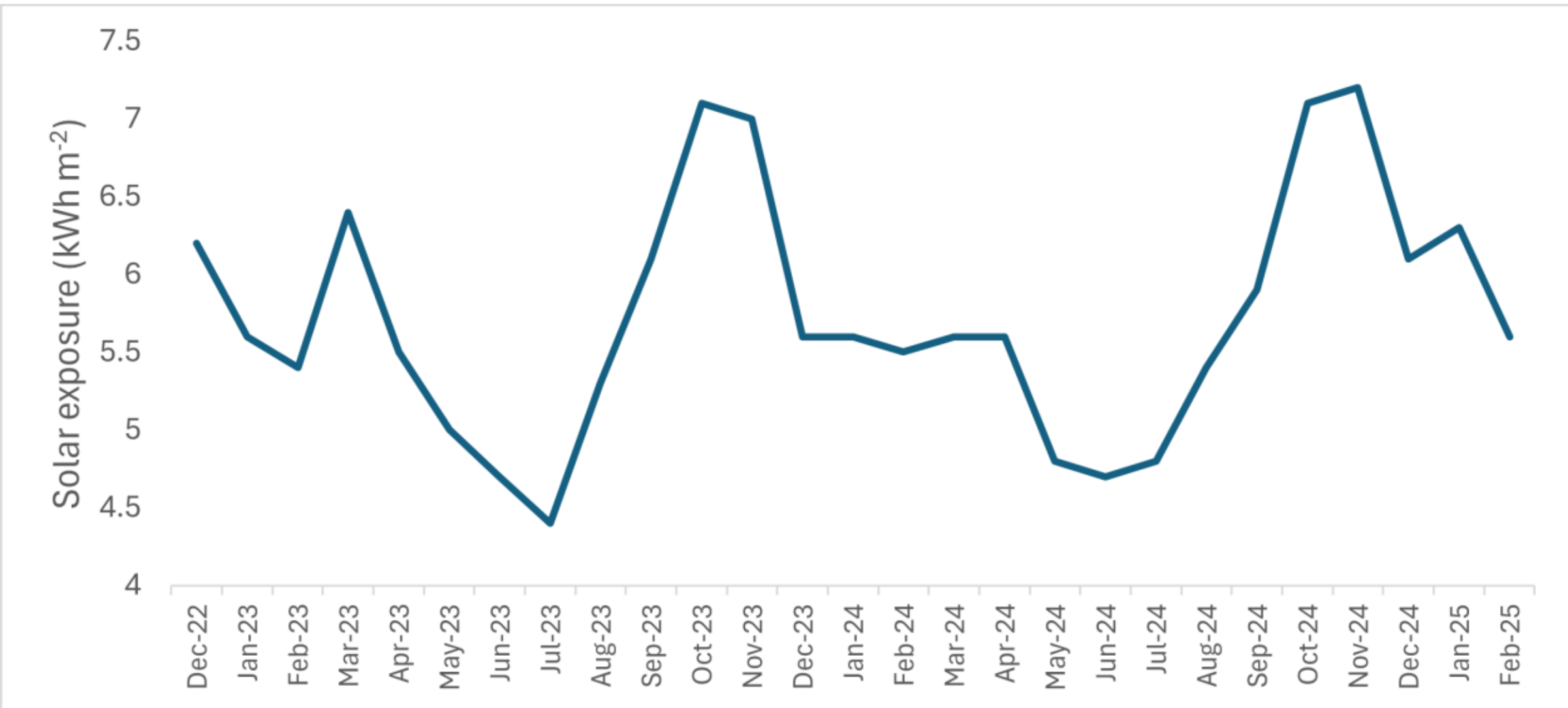

FIG S8. Monthly mean solar exposure recorded at the site across the study period (December 2022 to February 2025) encompassing both growing seasons (2023-2024 and 2024-2025). Data were obtained from the Australian Bureau of Meteorology station at Walkamin Research Station (station ID 31108).
